# Development of a Framework for the Implementation of Synchronous Digital Mental Health: Realist Synthesis of Systematic Reviews

**DOI:** 10.2196/34760

**Published:** 2022-03-29

**Authors:** David Villarreal-Zegarra, Christoper A Alarcon-Ruiz, GJ Melendez-Torres, Roberto Torres-Puente, Alba Navarro-Flores, Victoria Cavero, Juan Ambrosio-Melgarejo, Jefferson Rojas-Vargas, Guillermo Almeida, Leonardo Albitres-Flores, Alejandra B Romero-Cabrera, Jeff Huarcaya-Victoria

**Affiliations:** 1 Escuela de Medicina Universidad César Vallejo Trujillo Peru; 2 Dirección de Investigación, Desarrollo e Innovación Instituto Peruano de Orientación Psicológica Lima Peru; 3 Unidad de Investigación para la Generación y Síntesis de Evidencias en Salud Universidad San Ignacio de Loyola Lima Peru; 4 Peninsula Technology Assessment Group College of Medicine and Health University of Exeter Devon United Kingdom; 5 Georg-August-University Göttingen International Max Planck Research School for Neurosciences Göttingen Germany; 6 Center of Excellence in Chronic Diseases Universidad Peruana Cayetano Heredia Lima Peru; 7 Facultad de Psicología Universidad Nacional Mayor de San Marcos Lima Peru; 8 Facultad de Medicina Universidad Nacional de Trujillo Trujillo Peru; 9 Carrera Profesional de Medicina Humana, Facultad de Ciencias de la Salud Universidad Científica del Sur Lima Peru; 10 Unidad de Psiquiatría de Enlace Departamento de Psiquiatría Hospital Nacional Guillermo Almenara Irigoyen Lima Peru; 11 Escuela Profesional de Medicina Humana Universidad Privada San Juan Bautista Filial Ica Peru

**Keywords:** telemedicine, digital health, internet-based intervention, mental health, mental disorders, systematic reviews, qualitative research, realist review, mHealth, eHealth, telehealth

## Abstract

**Background:**

The use of technologies has served to reduce gaps in access to treatment, and digital health interventions show promise in the care of mental health problems. However, to understand what and how these interventions work, it is imperative to document the aspects related to their challenging implementation.

**Objective:**

The aim of this study was to determine what evidence is available for synchronous digital mental health implementation and to develop a framework, informed by a realist review, to explain what makes digital mental health interventions work for people with mental health problems.

**Methods:**

The SPIDER (Sample, Phenomenon of Interest, Design, Evaluation, and Research type) framework was used to develop the following review question: What makes digital mental health interventions with a synchronous component work on people with mental health problems, including depression, anxiety, or stress, based on implementation, economic, quantitative, qualitative, and mixed methods studies? The MEDLINE, EBM Reviews, PsycINFO, EMBASE, SCOPUS, CINAHL Complete, and Web of Science databases were searched from January 1, 2015, to September 2020 with no language restriction. A Measurement Tool to Assess Systematic Reviews-2 (AMSTAR-2) was used to assess the risk of bias and Confidence in Evidence from Reviews of Qualitative Research (CERQual) was used to assess the confidence in cumulative evidence. Realist synthesis analysis allowed for developing a framework on the implementation of synchronous digital mental health using a grounded-theory approach with an emergent approach.

**Results:**

A total of 21 systematic reviews were included in the study. Among these, 90% (n=19) presented a critically low confidence level as assessed with AMSTAR-2. The realist synthesis allowed for the development of three hypotheses to identify the context and mechanisms in which these interventions achieve these outcomes: (1) these interventions reach populations otherwise unable to have access because they do not require the physical presence of the therapist nor the patient, thereby tackling geographic barriers posed by in-person therapy; (2) these interventions reach populations otherwise unable to have access because they can be successfully delivered by nonspecialists, which makes them more cost-effective to implement in health services; and (3) these interventions are acceptable and show good results in satisfaction because they require less need of disclosure and provide more privacy, comfortability, and participation, enabling the establishment of rapport with the therapist.

**Conclusions:**

We developed a framework with three hypotheses that explain what makes digital mental health interventions with a synchronous component work on people with mental health problems. Each hypothesis represents essential outcomes in the implementation process.

**Trial Registration:**

PROSPERO International Prospective Register of Systematic Reviews CRD42020203811; https://www.crd.york.ac.uk/prospero/display_record.php?ID=CRD42020203811

**International Registered Report Identifier (IRRID):**

RR2-10.12688/f1000research.27150.2

## Introduction

Mental health is in crisis globally and the COVID-19 pandemic has suddenly revealed the magnitude of this problem [1,2]. To minimize health care gaps, the use of digital technologies has been proposed to be able to provide specialized treatment to a greater number of people in places with limited resources and to those with difficult access [3-7]. These technologies have been very well received and served to complement or improve the effectiveness of treatments for various chronic diseases [6]. In addition, these digital interventions show great promise in the care of mental health problems [8-10].

With the undeniable contribution of technologies in mental health care, it is important to document the aspects related to their challenging implementation [11], such as adaptability, cost, complexity, external policies and incentives, compatibility, or general fit between the digital health intervention and the organization, among others [12]. These features provide understanding about how and what works in these interventions, and considering the complexity as challenges in the implementation of telemedicine can help to reveal the deficiencies and inequalities of health care systems worldwide [13].

Currently, there are different frameworks to guide the implementation process, including Expert Recommendations for Implementing Change (ERIC), Promoting Action on Research Implementation in Health Services (PARIHS), or Consolidated Framework for Implementation Research (CFIR) [14,15]. However, we have not been able to find studies that developed frameworks to explain what makes digital mental health interventions work, specify in which contexts these digital interventions can be implemented, identify the mechanisms that facilitate or hinder their implementation, and elucidate the most important outcomes within the implementation process.

Despite not developing such a framework, previous studies have identified critical aspects to consider within the implementation process, such as the effectiveness of digital mental health interventions [16,17], barriers and facilitators to the implementation of electronic mental health interventions [18], or long-term cost-effectiveness studies [19]. However, this evidence alone is not sufficient to warrant the implementation of these interventions or their adoption by health systems [4].

The problem with not having a specific framework for the implementation of interventions focused on digital mental health is that this type of intervention has particular nuances compared with other types of health interventions [4], especially in low- or middle-income countries. More qualitative and flexible approaches are needed to understand the complexity of these interventions and what key elements could help their implementation [4]. Thus, the aim of this study was to determine what evidence is available for synchronous digital mental health implementation and to develop a framework, informed by a realist review, to explain what makes digital mental health interventions work for people with mental health problems.

## Methods

### Research Question

This systematic review adhered to the PRISMA (Preferred Reporting Items for Systematic Reviews and Meta-Analyses) guidelines [20]; a completed PRISMA checklist can be found in Multimedia Appendix 1. The detailed methodology is available in the published study protocol [21], and the study was registered in PROSPERO (CRD420203811). The SPIDER framework was used to develop the review question, which is based on describing the Sample (S), Phenomenon of Interest (PI), Design (D), Evaluation (E), and Research type (R) [22] (see Textbox 1).

**Research question development based on the SPIDER framework.**

- Sample

Adults with depression (or major depressive disorder), anxiety (or generalized anxiety disorder), stress (or trauma-related disorders), and/or general mental health problems (unspecified). Participants may be diagnosed through clinical interviews or categorized based on screening assessments (self-reported scales).

- Phenomenon of Interest

Any digital mental health intervention that includes a synchronous component, namely communication with a mental health professional (eg, psychiatrist, psychologist) or a health professional trained in mental health. These interventions included, among others, remote consultation, interactive application, video chats, and calls.

- Design

Systematic review.

- Evaluation

We included all types of outcomes of interest assessed by implementation studies, economic, qualitative, quantitative, and other study designs, including (1) health effectiveness outcomes (eg, depression, anxiety and/or stress symptoms, adherence to treatment), (2) patient outcomes (eg, quality of life, satisfaction), (3) economic outcomes, and (4) damage or adverse effects.

- Research type

Quantitative, qualitative, and mixed methods.

### Eligibility Criteria

#### Inclusion Criteria

Systematic reviews were selected that reported on inclusion/exclusion criteria for their included studies; conducted an adequate systematic literature search using at least two databases; and synthesized, assessed the quality of, and presented sufficient detail on their individual primary included studies [23]. The reviews had to include primary studies as a unit of analysis focused on a research question. We selected a publication start date of January 1, 2015, without language restrictions. We selected this time frame to include only the latest systematic reviews, since in the field of digital health, the launch of new technologies makes scientific development dynamic. Articles were also included if the primary studies in the review focused on adults with common mental health problems, defined as (1) adults with depression (or major depressive disorder), anxiety (or generalized anxiety disorder), stress (or trauma-related disorders), and/or general mental health problems (unspecified); or (2) adults attending an outpatient mental health consultation. The final inclusion criterion was that at least 90% of the primary studies assessed synchronous digital mental health or the results only for synchronous digital mental health are presented separately.

#### Exclusion Criteria

Narrative reviews, scoping reviews, primary studies, opinion/editorial manuscripts, letters to the editor, and reviews of mobile health intervention repositories (ie, app stores) were excluded. In addition, reviews that included primary studies of (1) adult participants with some other specific mental health condition outside of those listed above, (2) healthy adult participants (without mental health conditions), (3) adult participants receiving emergency/crisis psychiatric care, (4) interventions that lack a synchronic component (real-time information exchange between the user and mental health professional using technologies) or were not sufficiently clear of having a synchronic component, or (5) women with depressive postpartum symptoms were also excluded from the analysis.

### Information Sources

We searched the MEDLINE (Ovid), EBM Reviews (Ovid), PsycINFO (Ovid), EMBASE (Elsevier), SCOPUS, CINAHL Complete (EBSCOhost), and Web of Science databases, including Science Citation Index Expanded, Social Sciences Citation Index, and Conference Proceedings Citation Index (Clarivate Analytics). Articles published in the last 5 years (January 1, 2015, to April 30, 2020) were included with no language restrictions. The search of the databases was performed on April 30, 2020.

### Search Strategy

The search formula was created using thesaurus and entry terms for the following syntaxis: “telemedicine” AND “mental health, anxiety, depression or stress” AND “systematic reviews.” The full search strategy for each database is available in Multimedia Appendix 2.

### Study Records

#### Data Management

The records retrieved after the search were managed using the Rayyan QCRI free online application (eliminate duplicates, and review titles and abstracts) [24]. Full-text review and data extraction were performed in an Excel template.

#### Selection Process

The records were screened by title and abstract and then by full-text assessment. The records were divided into three groups with each consisting of a pair of independent reviewers (six people in total). Before conducting the review of the records, a calibration process was carried out, which was based on a pilot review of 30 registries and identifying that there was a discrepancy of less than 5% in the decision of whether or not to include the studies. During the review, in case of discrepancies between decisions within groups, peers discussed the discrepancies to reach an agreement. When it was not possible to reach an agreement among the peers, a third reviewer was included if necessary.

#### Data Collection Process

For each eligible study, data were extracted independently and duplicated on predesigned extraction forms. Reviewers solved discrepancies and a third reviewer evaluated any unresolved disagreement.

### Data Items

An extraction form was created for the included systematic reviews. We collected the following information: first author and publication date of the study, characteristics of the participants, main objective, research questions, inclusion criteria for the systematic review, search date, study selection process, quality assessment (if any), main findings, and limitations. The full text of the included articles, tables, and supplementary material were also gathered to perform the qualitative analysis of the text.

### Outcomes and Prioritization

The aim of our study was to perform a realist review of systematic reviews using a qualitative strategy to synthesize the information and answer our research question. Therefore, we did not look for a specific result such as effectiveness, cost-effectiveness, or similar. Instead, we were interested in identifying the full text of all studies that answered our research question to perform a grounded-theory analysis with an emergent approach [25]. Priority was given in the analysis to studies with the lowest risk of bias assessed.

### Risk of Bias in Individual Studies

To assess the quality of the included systematic reviews, we used A Measurement Tool to Assess Systematic Reviews-2 (AMSTAR-2), which has 16 domains. Seven of these domains are considered critical: (1) protocol registered before the start of the review, (2) adequacy of the literature search, (3) justification for the exclusion of individual studies, (4) risk of bias of individual studies included in the review, (5) adequacy of meta-analytic methods, (6) consideration of the risk of bias in interpreting the results of the review, and (7) assessment of the presence and likely impact of publication bias [26].

AMSTAR-2 classifies the quality of systematic reviews into four categories: high (none or one noncritical weakness), moderate (more than one noncritical weakness), low (one critical weakness with or without noncritical weaknesses), and very low (more than one critical weakness with or without noncritical weaknesses). The quality assessment was rated by two trained researchers independently. In case of difference in the overall quality assessment of the systematic reviews, the AMSTAR-2 criteria were discussed between the two researchers to reach a consensus.

### Data Synthesis

We developed a framework informed by a realist analysis of synchronous digital mental health interventions using a grounded-theory approach with an emergent approach [27]. The realist synthesis was based on interpreting, integrating, and inferring the evaluation elements to better understand the implementation of synchronous digital mental health interventions from all of the included studies [28]. To answer the question “what makes the implementation of these interventions work?”, hypotheses supported by the included studies’ results were developed and generated through discussion and consensus among the researchers [28]. Since our study was designed to perform a realist synthesis of the evidence, we focused on different outcomes to use them as input for assessing the implementation of synchronous digital mental health interventions. Therefore, we did not perform a quantitative synthesis in any case (ie, a meta-analysis of effectiveness).

Three researchers followed the three steps established by Thomas and Harden [28] for qualitative syntheses [29]. First, the extracted data were freely coded. The researchers read the full texts of the included articles and coded each text fragment that provided information to answer the research question. Second, the codified data were organized and then grouped based on descriptive aspects using a context-linked causality approach represented as “context+mechanism=outcome” [25]. Finally, the analytical concepts generated in the previous step were grouped so that they were related to each other. The elements that were related to each other were assumed to be part of a hypothesis that would help to answer the research aim.

The selection of the studies for the realist review was based on the AMSTAR-2 score, with the highest-quality studies being assessed first. We assessed all included studies, down to the criterion of theoretical saturation [30]. All qualitative analyses were performed with NVivo software (version 12, QSR International).

### Confidence in Cumulative Evidence

The Confidence in Evidence from Reviews of Qualitative Research (CERQual) approach, which has four components (Methodological Limitations, Relevance, Coherence, and Appropriateness Data), was assessed by a researcher and then reviewed by another independent researcher. The CERQual was evaluated to contribute to an overall assessment of each hypothesis resulting from the realist synthesis to determine the level of confidence (high, moderate, low, or very low) and to present the overall assessment in a Summary of Qualitative Findings table [31,32].

## Results

### Study Selection

**Figure 1:**
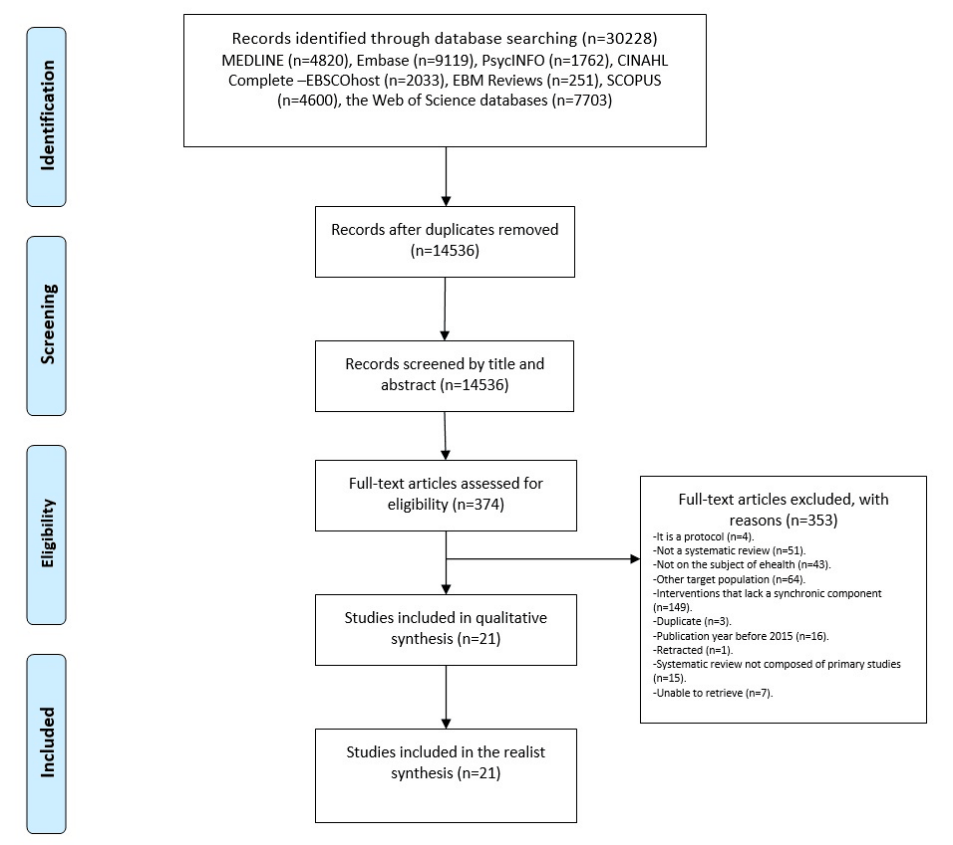
Flowchart of the study selection process.

The search strategy retrieved 30,228 records, and after duplicated cleaning, we obtained 14,536 unique records. The evaluation by title and abstract identified 374 results that were evaluated at the full-text level. Among those, 353 were excluded. The reasons for exclusion are listed in Multimedia Appendix 3. Finally, 21 systematic reviews were included in this study (see Figure 1).

### Study Characteristics

The included systematic reviews analyzed a median of 27 studies (range 9-155). Eleven studies reported some form of synchronous digital mental health intervention based on internet, telephone, or online cognitive behavioral therapy (CBT) as the primary intervention [19,33-42]. The remaining studies reported a mix of digital mental health interventions based on synchronous components (ie, telephone, videoconferencing) and asynchronous components (ie, text messages, email, chats, instructional videos, podcasts). Most of the systematic reviews included exclusively randomized controlled trials (RCTs) as primary studies, two included only non-RCTs, and five studies included both. Only six studies did not include a meta-analysis. With respect to the type of therapy, nine reviews stated CBT as the target therapy, one review used the transdiagnosis method, and one included mindfulness-based interventions. The individual characteristics of the included studies are presented in Multimedia Appendix 4. It is important to mention that despite having no language restrictions, all of the included articles were published in English and the systematic reviews did not include qualitative studies.

### Risk of Bias Within Studies

Most of the studies (19/21, 90%) of the included systematic reviews performed a risk of bias assessment. The most commonly used instrument was the Risk of Bias Cochrane Collaboration tool (12/24, 57%) [33-35,37,38,40,43-48]. Seven studies used other tools to assess the risk of bias such as the Effective Public Health Practice Project Quality Assessment Tool (2/21, 10%); Grading of Recommendations Assessment, Development and Evaluation (1/21, 5%); and others. Only two studies did not report using any risk of bias tool [41,42]. Ten studies did not appropriately account for the risk of bias of the individual studies included when interpreting the results of their review.

**Figure 2:**
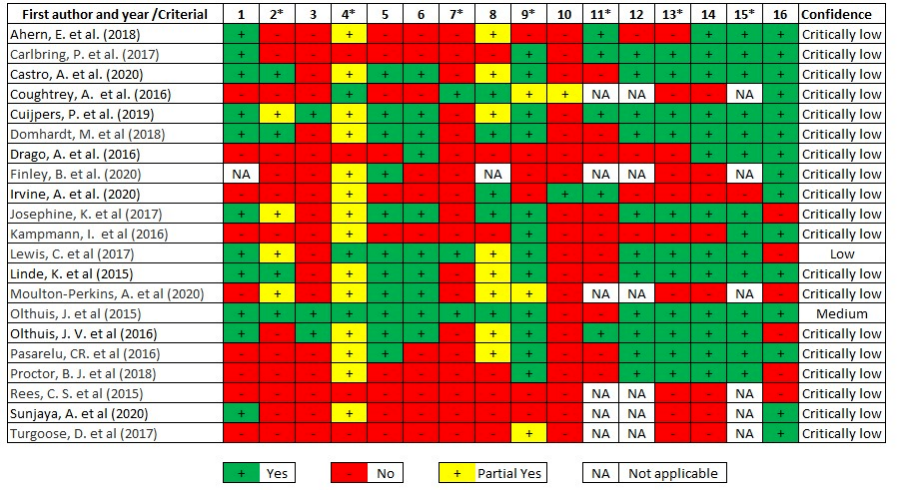
Risk of bias assessment of individual studies, according to AMSTAR-2. 1: Did the research questions and inclusion criteria for the review include the components of PICO (Population, Intervention, Control, Outcomes)? *2: Did the report of the review contain an explicit statement that the review methods were established prior to conduct of the review and did the report justify any significant deviations from the protocol? (critical item); 3: Did the review authors explain their selection of the study designs for inclusion in the review? *4: Did the review authors use a comprehensive literature search strategy? (critical item); 5: Did the review authors perform study selection in duplicate? 6: Did the review authors perform data extraction in duplicate? *7: Did the review authors provide a list of excluded studies and justify the exclusions? (critical item); 8: Did the review authors describe the included studies in adequate detail? *9: Did the review authors use a satisfactory technique for assessing the risk of bias (RoB) in individual studies that were included in the review? (critical item); 10: Did the review authors report on the sources of funding for the studies included in the review? *11: If meta-analysis was justified, did the review authors use appropriate methods for statistical combination of results? (critical item); 12: If meta-analysis was performed, did the review authors assess the potential impact of RoB in individual studies on the results of the meta-analysis or other evidence synthesis? *13: Did the review authors account for RoB in individual studies when interpreting/discussing the results of the review? (critical item); 14: Did the review authors provide a satisfactory explanation for, and discussion of, any heterogeneity observed in the results of the review? *15: If they performed quantitative synthesis, did the review authors carry out an adequate investigation of publication bias (small study bias) and discuss its likely impact on the results of the review? (critical item); 16: Conflict of interest declaration.

Olthuis et al [47] presented a medium level of confidence and Lewis et al [38] presented low confidence. The rest of the included systematic reviews presented a critically low level of confidence (see Figure 2). On average, the included reviews only met 40% of the AMSTAR-2 risk of bias items. The reviews included in Rees et al [41] failed to accomplish any of the AMSTAR-2 items and those in the study by Turgoose et al [49] only passed one AMSTAR-2 item.

The AMSTAR-2 items that were the most fulfilled (if applicable) were item 15 (critical) assessing the presence and likely impact of publication bias (14/21, 93%) and item 12 (noncritical) assessing the potential impact of risk of bias in individual studies (11/21, 73%) in the case of meta-analysis. The AMSTAR-2 items that were the least fulfilled were item 10 (noncritical) on whether the review reported the funding sources of the included studies. Only the study by Irvine et al [36] achieved compliance. Two other items that had a low compliance rate (3/21, 14%) were item 4 (critical) on the adequate literature search and item 3 (noncritical) on the justification for the decision on the study designs to be included in the review, and only one study met each of these criteria [46].

### Realist Synthesis

#### Overview

**Figure 3:**
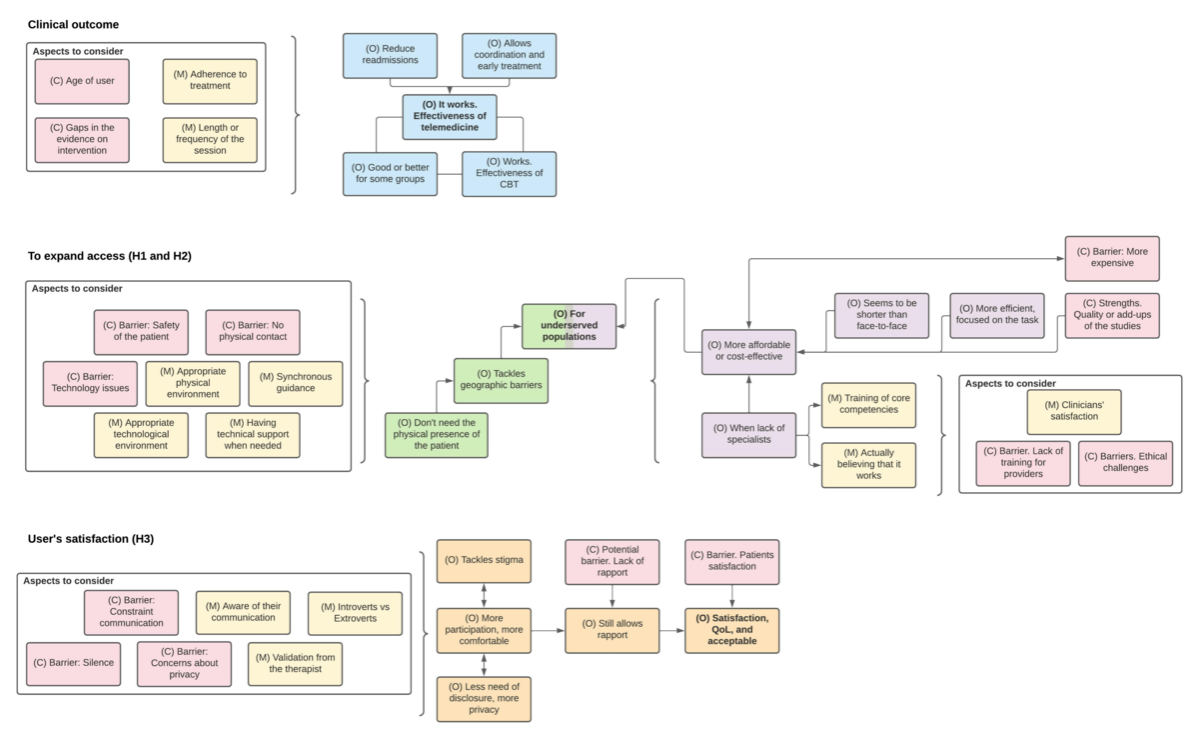
Results of the three hypotheses (H1-H3) of the realist synthesis. C: context (pink); M: mechanism (yellow); O: outcome (different colors for each hypothesis); CBT: cognitive behavioral therapy; QoL: quality of life.

Synchronous digital mental health interventions provide effective clinical outcomes (see Figure 3). Some systematic reviews identified that digital mental health interventions based on CBT (ie, telephone, internet-based, videoconferencing, online) were equally effective as face-to-face CBT in the treatment of specific mental health conditions (eg, social anxiety disorder, posttraumatic stress disorder [PTSD], panic, depressive symptoms, body dissatisfaction, insomnia, specific phobias) [33-36,42,47,49,50]. In addition, the different theoretical models used in CBT-based digital mental health interventions (ie, classical, mindfulness, transdiagnostic, nonspecific) and nonspecific digital mental health interventions had a moderate to large effect in reducing depressive, anxious, and PTSD symptoms compared to control situations [35,37-40,44-46,48,49,51]. Furthermore, different formats of individual and group electronic interventions (ie, telephone, videoconferencing) and guided self-help treatment had comparable effectiveness in depression and anxiety treatment [35,45,46]. In addition, digital interventions have shown to be effective in different population groups such as adults and elder people [33,34,40,45], veterans [47,49], and people with multiple sclerosis [48].

The advantages of interventions using technology are allowing the inclusion of add-ups to the therapy (eg, written, audio or visual materials to access online or download, diary-keeping, chats [19], emails [19,47], online forums [19,43,46], new or existing platforms such as Skype or Zoom) [49]. These interventions also promote better coordination of care and early treatment [42,49].

Guided synchronous components are essential elements in digital interventions to reduce anxiety. They are more effective and significantly improve adherence compared to unguided interventions or those with only asynchronous components [43]. It is also unclear which guided synchronous components are the most effective or whether there are cumulative effects when combining them [43]. Of note, CBT-based and heterogeneous digital mental health interventions (not CBT-based) showed no difference in their effectiveness in reducing PTSD symptoms [38].

Three main hypotheses were derived from this analysis, which are summarized in Textbox 2 and described in detail in the following sections.

**Hypotheses for why digital mental health interventions work for people with mental health problems based on the realist synthesis of reviews.**

**Hypothesis 1**

Synchronous digital mental health interventions reach populations otherwise unable to have access through face-to-face interventions, since they do not require the physical presence of the therapist nor the patient, thereby tackling geographic barriers posed by in-person therapy (to expand access).

**Hypothesis 2**

Synchronous digital mental health interventions reach populations otherwise unable to have access via face-to-face interventions because they can be successfully delivered by nonspecialists, which makes them more cost-effective to implement in health services (to expand access).

**Hypothesis 3**

Synchronous digital mental health interventions are acceptable by patients and show good results in satisfaction, because they require less need of disclosure and provide more privacy, comfortability, and participation, enabling the establishment of rapport with the therapist (user satisfaction).

#### Hypothesis 1

Synchronous digital interventions in mental health reach populations that would not have access through face-to-face interventions, such as children, veterans, refugees, and people living in rural areas [50,52]. This is because these interventions do not require the physical presence of both the patient and the therapist (see Figure 3). We also found that these interventions can reduce geographical barriers to access (eg, mobilization for several hours). In addition, they can interact in real time [41] and tackle the geographic barriers of travel required to receive care, thereby being accessible even from remote areas [37,41,42,46-48,50].

Some aspects need to be taken into consideration for the delivery of successful therapy through synchronous digital mental health interventions. The first is to find a quiet area in the home or at the usual environment of the patient to receive the session, which could represent a challenge for many [49]. The second aspect is that the platform should be as stable as possible since ineffective internet service could lead to withdrawing the therapy [49], and the quality of the image and sound could be associated with satisfaction [39]. Third, the possibility to expand the use of telepsychiatry will require the development or improvement of a software specially designed for that purpose [42]. Finally, the presence of technical support when needed should be considered, as one systematic review found that scheduled guidance showed better outcomes on anxiety symptom severity at postintervention and follow-up [43].

The presence of synchronous human support seems to improve the delivery of digital mental health sessions, although the evidence is not conclusive [19,43]. Guided interventions were superior to completely unguided interventions for symptom severity across mental disorders and presented higher treatment adherence [43]. In studies that used local clinics rather than home-based teletherapy, it was recommended to have local staﬀ on hand to assist, such as to receive homework and other materials via fax machine and disseminate them to participants [42]. However, in the future, artificial intelligence could replace human support to generate computer responses [33].

Additionally, we found some barriers. The first barrier is the absence of physical contact. One review identified that patients receiving in-person treatment were more likely to complete the home assessments and tasks given [49]. The second barrier is that the safety of the patient could be compromised. It is worth noting a potential issue with interventions using technology. The distance between the patient and therapist could put patients’ safety at risk, as they may not receive the necessary care in the event of a crisis or emergency [43]. Some studies also suggested the presence of an extra person to provide in-person support in case of emergencies [43,49], although not all studies showed favorable results [19,33,43]. Finally, the presence of technical issues could impose a potentially modifiable barrier. Some flaws found during the therapy delivery were limited connectivity, the lack of human resources and telepsychiatry equipment [42], low image resolution, difficulties for establishing the connection, slight audio delays, and problems with the internet connection [42]. Moreover, a systematic review assessing mindfulness-based cognitive therapy for stress reduction found that the users’ dissatisfaction was linked to technical issues [39].

#### Hypothesis 2

A second reason for why these interventions reach populations that otherwise would not have access to face-to-face interventions is that they are an accessible and cost-effective treatment in the short term [19]. This may lead to reductions in mental health costs, at least in depression [19]. It should be noted that CBT-based digital interventions tend to be slightly more expensive compared to usual treatment at baseline. This is because their cost-effectiveness improves when considering their positive effect on quality-adjusted life years [19] and their costs in the long-term, since they require limited interaction between the patient and therapist [34,42].

This higher cost-effectiveness is associated with different components. Regarding phone sessions, they adhere to a more structured format and focus on problem-solving and tasks, resulting in more efficient and direct sessions [36] with shorter durations than in-person therapies [34,36]. It should be noted that the session duration of these interventions was not associated with better outcomes in cases of anxiety and depression, although the therapy duration varied from 19 to 150 minutes [40].

Evidence suggests that physicians, psychiatrists, psychologists, or nurses trained for various mental health problems could perform digital interventions such as telepsychiatry or teleconsultations [52]. This enables optimization for using available human resources when there is a reduced number of specialists for large populations, since nonspecialists with adequate training and supervision are as effective as specialists for this purpose [41,43]. For this outcome, it is important to consider some barriers. A potential barrier was the provision of care by nonspecialists, highlighting the importance of having appropriate training and supervision to provide long-distance care. Training for therapists providing interventions using technology should include content on good clinical practices [39,52], the use of technology [49] and telepsychiatry [52], the management of risk or crises [43], as well as potential ethical and/or legal conflicts [50]. Another potential barrier is distrust of the health personnel. One study pointed out that therapists showed greater preference for face-to-face interventions compared to online interventions [38], while another found that some professionals may be reluctant to apply electronic interventions using telephones to treat mental health problems, arguing that it could harm the interactions with the user [36]. However, evidence suggests that the use of electronic interventions with telephones does not change interaction patterns in consultations (duration, alliance, disclosure, empathy, attention, and participation) [36].

Some relevant aspects to consider are clinicians’ satisfaction, the lack of training for providers, and ethical challenges. For example, a systematic review of teletherapy for veterans with PTSD found high fidelity to the intervention and good therapist competence, as well high levels of satisfaction among clinicians in terms of their confidence for the delivery of these forms of therapies [49]. However, as mentioned above, proper training is needed for successful delivery [39,49,52], and the ethical and legal aspects should be established [50].

#### Hypothesis 3

Telepsychiatry for patients with PTSD shows the advantage of diminishing the risk of stigmatization. Since patients are treated from their own homes and are no longer required to visit a psychiatric facility, they feel more motivated to seek mental health care [42]. One systematic review found that patients exhibited more active participation at distance-delivered therapies compared to face-to-face interviews. This may be due to the feeling of “safety” that being at a different location from the therapist could produce. They found that neither empathy, attention, nor participation diminished when using telephone interventions [49]. Additionally, telephonic interventions offer the patient a potentially immediate, anonymous, and easy-to-access option [34]. Other authors pointed out that patients felt that the therapist could understand them better during face-to-face therapies. However, there were no differences in the ability of the therapist to guide the patients to “open themselves” between modalities [36]. It was reported that the efficacy of interventions was similar across modalities and although the interaction between patient and therapist was lower [39], the therapeutic alliance was able to be achieved without limitations [42,47], except for the difficulties at reading corporal language [49].

Telephone and video call interventions were usually acceptable and efficient for digital mental health [41]. This is probably because more access to care was allowed for children and adults with comorbid psychiatric and complex medical illnesses in various settings, age spans, and demographic characteristics, including rural areas [52]. Although there is greater satisfaction on the users’ side (and therefore an improvement in mood state), this does not imply that there are improvements in the quality of life, since recovery (the relief of depressive symptoms) does not necessarily amount to parallel improvements in quality-of-life measures [19]. In addition, it should be considered that these two outcomes do not follow the same recovery rate.

It is also worth noting that during telephone therapies, the patients could develop an awareness of their own emotional and affective changes by listening to their own voice. Moreover, since there is no difference in the measure of how “closely” the therapist could be listening as in usual face-to-face communication, the patients could more easily feel the “connection” with their therapist and enhance disclosure of feelings and emotions [36]. It was found that the use of technology did not influence the therapeutic alliance with their patients [39,47,49]. This could be explained by the fact that, in this context, the therapist’s validation is not based on nonverbal communication but rather by their listening capacity, their verbal clarity, their tone of voice, and how the patient experiences it [43]. Indeed, telephone therapy could work better for introverted patients because it provides more anonymity, creating a sense of safety [34,43].

Some aspects to consider include barriers such as awkward silence, concerns about privacy, and constrained communication. Some patients had expressed their privacy concerns. For instance, veterans with PTSD mentioned questions about the confidentiality of the video transmissions and the data they shared during the consultation [49]. In that same review, constrained communication for detecting body language and nonverbal communication by clinicians when conducting teletherapy for veterans with PTSD was reported. However, they could still develop rapport [49]. Finally, during communications where there is no video of the patient, as in telephone therapy, silences during the patients’ speech were more challenging to interpret [36].

One review noted that only two studies reported providing ongoing technical support during interventions [39]. In addition, none of the studies included in their review mentioned videoconferencing-specific good practice guidelines, training of facilitators to conduct online psychological interventions, or contingency plans to support remote participants [39]. Moreover, few studies reported on the frequency of technical problems [39].

#### Gaps: Limitations of Digital Mental Health Reported in Reviews

Lastly, even though technology interventions have proven to be as effective as in-person sessions and have a 2.13-times higher probability of achieving an appointment once a month [52], some limitations should be noted. First, their effectiveness will depend on treatment adherence [40]. Second, there is limited information on whether CBT-based electronic interventions maintain their beneficial effects over time; two systematic reviews did not identify sufficient evidence to support the benefits of this therapy at 3 or 6 months posttreatment for PTSD cases [38,47]. Third, most of these studies did not use randomization and their sample sizes were small; therefore, more research is needed [19,35-37,39,41,44-46,48,49,51]. Finally, most of the available evidence comes from high-resource countries with integrated health systems and larger research budgets [42]. Hence, some results may not be extrapolated to low- or middle-income countries.

### Confidence in Cumulative Evidence

An overall analysis of the CERQual assessment showed that the hypotheses presented have low or very low confidence in the evidence (see Multimedia Appendix 5). The main methodological limitations are that the studies come from research with a low or very low confidence level. In terms of coherence, the baseline assumption and hypothesis 1 showed adequate coherence between the different findings, whereas hypotheses 2 and 3 showed moderate concern, since some reviews have heterogeneous results. Finally, all hypotheses showed the adequacy of the data and relevance of the results.

## Discussion

### Main Findings and Interpretation

Our study developed a framework based on three hypotheses and a baseline assumption to understand/explain the implementation of synchronous digital mental health interventions. From the 21 systematic reviews included, studies showed that synchronous digital mental health interventions provide effective clinical outcomes and are as effective as face-to-face therapies that address mental health conditions [33-36,42,47,49,50]. These digital interventions reach populations such as children, veterans, refugees, and people living in rural areas [50,52], thereby reducing geographical barriers to access. Moreover, since patients are treated from their own homes and are no longer required to visit a psychiatric facility, this can reduce the fear of mental health stigma [39]. Nevertheless, there are few considerations to achieve successful therapy, such as a quiet environment for the patient to receive the session, a stable platform [49], the development or improvement of a software specifically designed for that purpose [42], and the presence of technical support when needed [43]. Some limitations should be noted due to the critically low level of confidence presented in the studies and the fact that most of the available evidence comes from high-resource countries with integrated health systems and larger research budgets [42]. Hence, some results may not be extrapolated to low- or middle-income countries.

### Comparison With Other Studies

Implementation science is an emerging and rapidly growing field that has established frameworks, methods, and strategies to improve the adoption and sustainability of interventions within the real world [53]; it has also identified different barriers and facilitators to the implementation of digital mental health interventions [53]. However, strategies specifically designed for implementing digital mental health interventions within the health care system are still limited [53-55].

The implementation of digital mental health interventions allows for overcoming many barriers in health access, such as geographic, human resources, and stigma barriers. These types of interventions allow patients and therapists to remain in their usual, more comfortable, or safer locations. Another advantage is that our framework supports that other mental health providers with lower degrees could deliver digital mental health interventions after appropriate training, which would increase the available human resources pool of therapists [41,43]. In addition, digital mental health interventions could be more attractive than face-to-face therapies, as they present the opportunity to increase privacy and minimize the risk of stigmatization since they can take place outside of mental health institutions, which is especially relevant for populations in which the presence of potential social stigma interferes with the decision to attend mental health facilities [42].

Our study provides hypotheses based on systematic reviews, which allow for obtaining a better understanding for the implementation of synchronous interventions in digital mental health. However, our framework does not provide specific steps or strategies to carry out the implementation process. Therefore, to fill this gap, other researchers could use the ERIC project framework, which presents four general phases for implementing digital interventions in the health system: an implementation strategy exploration phase, preparation phase, implementation phase, and sustainability phase [53,56]. It should be noted that other frameworks that systematize the implementation steps could be used to perform the implementation task, as long as they are adapted to the particularities of the context, health system, resources, and willingness of the actors involved. An alternative that has proven to be useful in favoring the implementation of interventions from heterogeneous contexts is a formative study design that allows for the contextualization of these interventions while evaluating their acceptability, efficiency, and safety within the health system or community [57]. However, this requires greater investment in research by low- and medium-resource countries.

There are currently no frameworks to explain the implementation of digital interventions as the main component in mental health care. Although we have not identified any studies directly comparable to ours, there are related studies. For example, a systematic review of barriers and facilitators to the implementation of electronic mental health interventions identified that the acceptability of electronic interventions depends on (1) patients’ and professionals’ expectations, (2) preferences about what they would receive and what they provide during care, and (3) the appropriateness of the electronic intervention to address patients’ mental health conditions [18]. One study proposed an ethical framework for the development, use, and implementation of digital mental health interventions such as chatbots, based on the principles of beneficence, nonmaleficence, autonomy, justice, and explicability [58]. Although chatbots are not synchronous interventions, they can be used as additional components in synchronous interventions. In the absence of an integrative framework, our study proposes a technical underpinning of available evidence to enable decision-makers to implement electronic interventions to address mental health. We identified different reviews supported by electronic interventions for anxiety, depression, and PTSD, which are equivalent to face-to-face interventions [33-36,42,47,49,50] and are cost-effective in the long term [19].

Despite evidence in favor of digital mental health interventions, there is a considerable difference between the reports from high-income and low-income countries. Some high-income countries had sufficient evidence to conduct country-focused effectiveness evaluations. For example, a systematic review from the United Kingdom identified 7 out of 48 digital interventions promoted by their health system for depression and anxiety as having a small but consistent effect and recommended their use [59]. In addition, the disparity in the amount of evidence remains in economic research, where a systematic review of economic studies identified that internet-based digital interventions for anxiety and depression are cost-effective and recommended their use; however, only studies from high-income countries were identified [60].

In contrast, no reviews of effectiveness, cost-effectiveness, or acceptability of electronic interventions were identified for low- and middle-income countries. The limited evidence from low- and middle-income countries suggests that their health systems made decisions based on minimal local evidence, low-quality evidence (ie, expert review), and evidence from only high-income countries (ie, different contexts). Additionally, material and economic resources and internet access are limited in low- and middle-income countries. Thus, sufficient internet access for health care providers and users should be assured for implementing these technologies. Other problems that could generate inequity, such as limited access to smartphones in rural and low-income areas, low internet speed, and network instability, could generate gaps for adequate implementation of these technologies.

An additional element to highlight, apart from the effectiveness or cost-effectiveness of electronic interventions, is the positive effects they could have on patients’ quality of life. Although quality of life was not an outcome in our study, we found evidence that electronic interventions to treat mental health positively affect quality of life [38,40,46]. These results are consistent with other systematic reviews showing that CBT-based interventions (eg, face-to-face, internet, or group) improve participants’ quality of life [61,62]. Furthermore, this secondary benefit of electronic mental health interventions on quality of life appeared to affect years of life lost due to disability [63]. This explains why this outcome is key for understanding the cost-effectiveness of this type of intervention since its long-term effect is to reduce costs within the health system [19].

### Implementation and Public Health Implications

Decision-makers and researchers could use this relevant information to support the implementation of electronic mental health interventions within their health systems (ie, teleconsultation network). There is evidence to support digital interventions due to their effectiveness in depression, anxiety, and PTSD; their feasibility and acceptability; their safety; and the additional effect on the quality of life of patients [35,37-40,44-46,48,49,51]. The treatment models that have the most empirical support are those based on CBT, which could be the first type of interventions to be implemented. In addition, evidence supports that models of CBT electronic interventions are cost-effective, making their implementation within health systems feasible in the long term.

Health systems must develop legislation and basic technological conditions to achieve the implementation of synchronous digital mental health interventions. First, legislation such as privacy policies, terms of use, and technological requirements of teleconsultation platforms should be established [4]. All of these issues should be covered and regulated by national policies and there should be an entity to enable their regulation. Consequently, health care systems should develop an integrated digital health/digital mental health system that is user-friendly for all literacy levels.

Second, there is a need for quality internet and cell phone services to increase the likelihood of adherence [4,39,42]. Collaboration among public and private sectors is needed in this regard. Technical support and access to therapies should be flexible in terms of schedules, since participants would adjust the delivery to their own timetables. Hence, night schedules should be considered. In addition, training for personnel with minor degrees must be guaranteed in a standardized and systematic way [41,43,52].

Third, for the implementation and use of electronic interventions, it is necessary to identify the barriers within each health system to achieve the acceptance of the different actors. Lack of access to technology (especially in low-resource countries), limited training in teleconsultation or reluctance of health personnel to use the technology, problems related to patient safety or privacy, and limited legislation on teleconsultation at the country level are necessary elements to evaluate during the planning of electronic interventions in mental health [64].

Fourth, the context of the COVID-19 pandemic has enhanced the use of technologies to provide health care and reduce health care access gaps, and decision-makers need to take advantage of this context to enhance the implementation and adoption of these types of interventions [3-7]. It should be noted that digital interventions are not only a short-term solution, as the trend is to incorporate them as a key part of cost-effective health care systems [19,34,42].

### Strengths and Limitations

One of the strengths of our study is that we collected information from systematic reviews in a large number of databases, assuring the comprehensiveness of the evidence included. However, our study has limitations. First, the quality of the systematic reviews included was critically low for the most part, which could limit the confidence in the conclusions of the study. Other studies have already reported the low quality of systematic reviews and clinical practice guidelines in mental health [65-67]. Second, the electronic interventions evaluated are very heterogeneous both in the form of delivery (ie, telephone, internet-based, videoconferencing, online) and in the theoretical models used (classical CBT, mindfulness-based CBT, transdiagnostic CBT, nonspecific). Therefore, there may be variations in effect, safety, and acceptability in the way of delivery and the theoretical model used. Third, most of the research has been conducted in high-income countries, and therefore the results may not be comparable in low- and-middle income countries. Fourth, although a realist review analysis was rigorously carried out, the evidence evaluated has methodological limitations, resulting in overall low certainty of the evidence.

### Conclusions

Our study assessed all available evidence for the implementation of synchronous digital mental health interventions and developed a framework for the implementation of synchronous digital mental health based on three hypotheses. Since it is known that digital mental health interventions are clinically effective, we hypothesized that those interventions reach otherwise inaccessible populations since they abolish the need of physical presence and mobilization (hypothesis 1) or because a nonspecialist could deliver it with the additional advantage of reducing expenses (hypothesis 2), and that digital interventions are acceptable for those receiving them and maintain the establishment of rapport (hypothesis 3). Each hypothesis represents important outcomes in the implementation process. In addition, we analyzed the barriers and facilitators for these outcomes and identified gaps in the body of evidence that require attention from future researchers.

Our study provides a framework to understand the implementation of synchronous digital mental health interventions, suggests elements to consider at the time of implementation, and establishes gaps. This information will guide decision-makers, researchers, health system managers, and implementation teams.

## Appendix

Multimedia Appendix 1 PRISMA checklist.

Multimedia Appendix 2 Search strategy.

Multimedia Appendix 3 Reasons for articles excluded.

Multimedia Appendix 4 Characteristics of the included studies.

Multimedia Appendix 5 Confidence in cumulative evidence assessed with the Confidence in Evidence from Reviews of Qualitative Research (CERQual) approach.

## Abbreviations

AMSTAR-2: A Measurement Tool to Assess Systematic Reviews-2
CBT: cognitive behavioral therapy
CERQual: Confidence in Evidence from Reviews of Qualitative Research
CFIR: Consolidated Framework for Implementation Research
ERIC: Expert Recommendations for Implementing Change
PARIHS: Promoting Action on Research Implementation in Health Services
PRISMA: Preferred Reporting Items for Systematic Reviews and Meta-Analyses
PTSD: posttraumatic stress disorder
RCT: randomized controlled trial
SPIDER: Sample, Phenomenon of Interest, Design, Evaluation, and Research type
