# Supplementary material for: Development of a Framework for the Implementation of Synchronous Digital Mental Health: Realist Synthesis of Systematic Reviews
Source: JMIR Ment Health. 2022 Mar 29;9(3):e34760. doi: 10.2196/34760 (PMC9006141; doi:10.2196/34760)
Supplement: Multimedia Appendix 2 [file mental_v9i3e34760_app2.docx]

**Search strategy.**

MEDLINE (4820), EBM reviews (251), and PsycINFO (1762) (by Ovid)

| N° | Search strategy | Records |
| --- | --- | --- |
| #1 | ("Telemedicine" or "digital health" or Telehealth or mHealth or "Mobile Health" or eHealth or e-Health or "Electronic health" or emedicine or e-medicine or "Electronic medicine" or "Remote Consultation" or Teleconsultation* or multimedia or apps or facebook* or twitter* or tweet* or mobile* or phone* or "social media" or ios or android or ipad* or iphone* or ipod* or tablet* or computer* or online or web or "personal digital assistant" or chat or eTherap* or gaming or iCBT or "information technolog*" or "instant messag*" or internet* or "social network*" or "social medi*" or teletherap* or virtual*).ti,ab. OR Telemedicine/ or Remote Consultation/ or Distance Counseling/ or Social Media/ or Internet/ or Social Networking/ | 1.390.804 |
| #2 | ("Mental Health" or "Mental Disorders" or "Mental problem*" or "Mental Disorder" or "Behavior Disorders" or "Behavior Disorder" or "Psychiatric disease" or "Psychiatric diseases" or "Psychiatric illness" or "Psychiatric illnesses" or "Psychiatric disorder" or "Psychiatric disorders" or "Mood disorder" or "Mood disorders" or "psychological problem" or "psychological distress" or "psychological stress*" or "psychological disturb*" or "psychological ill*" or "psychological disease*" or "emotional problem*" or "emotional distress" or "emotional stress*").ti,ab. OR Mental Health/ or Mental Disorders/ or Mood Disorders/ or Stress, Psychological/ or Psychological Distress/ | 896.077 |
| #3 | ("Depression" or "Depressive Disorder" or Depressi* or "affective disorder" or "affective disorders" or "mood disorder" or "mood disorders" or dysphori* or dysthymi* or melancholia* or Anxiety Disorders or anxiet* or Hypervigilance or Nervousness or Agoraphobia or Catastrophiz* or Phobi* or Panic* or Stress* or Distress* or trauma* or Post-Traumatic or Posttraumatic or Emotional Adjustment or "General disorder" or "Emotional Adaptation" or "Psychological Adjustment" or "Psychologic Adaptation" or "Psychological Adaptation" or "Adaptive Behavior").ti,ab. OR Depression/ or Depressive Disorder/ or Anxiety Disorders/ or Anxiety/ or Agoraphobia/ or Phobic Disorders/ or Panic/ or Stress Disorders, Traumatic/ or Stress Disorders, Post-Traumatic/ or Stress, Psychological/ or Adaptation, Psychological/ or Psychological Distress/ or General Adaptation Syndrome/ or Adjustment Disorders/ | 2.774.116 |
| #4 | ("meta analysis" OR "meta-analysis" OR metanalysis OR metaanalysis OR "Systematic review" OR "Systematic reviews" OR "meta synthesis" OR metasynthesis).ti,ab. OR Systematic Review/ OR Meta-Analysis/ | 375.681 |
| #5 | 2 or 3 | 3.223.448 |
| #6 | 1 and 4 and 5 | 8803 |
| #7 | limit 6 to yr="2015 -Current" [Limit not valid in DARE; records were retained] | 6833 |

EMBASE (Elsevier)

| N° | Search strategy |  |
| --- | --- | --- |
| #1 | 'telemedicine':ti,ab,kw OR 'digital health':ti,ab,kw OR telehealth:ti,ab,kw OR mhealth:ti,ab,kw OR 'mobile health':ti,ab,kw OR ehealth:ti,ab,kw OR 'e health':ti,ab,kw OR 'electronic health':ti,ab,kw OR emedicine:ti,ab,kw OR 'e medicine':ti,ab,kw OR 'electronic medicine':ti,ab,kw OR 'remote consultation':ti,ab,kw OR teleconsultation*:ti,ab,kw OR multimedia:ti,ab,kw OR apps:ti,ab,kw OR facebook*:ti,ab,kw OR twitter*:ti,ab,kw OR tweet*:ti,ab,kw OR mobile*:ti,ab,kw OR phone*:ti,ab,kw OR 'social media':ti,ab,kw OR ios:ti,ab,kw OR android:ti,ab,kw OR ipad*:ti,ab,kw OR iphone*:ti,ab,kw OR ipod*:ti,ab,kw OR tablet*:ti,ab,kw OR computer*:ti,ab,kw OR online:ti,ab,kw OR web:ti,ab,kw OR 'personal digital assistant':ti,ab,kw OR chat:ti,ab,kw OR etherap*:ti,ab,kw OR gaming:ti,ab,kw OR icbt:ti,ab,kw OR 'information technolog*':ti,ab,kw OR 'instant messag*':ti,ab,kw OR internet*:ti,ab,kw OR 'social network*':ti,ab,kw OR 'social medi*':ti,ab,kw OR teletherap*:ti,ab,kw OR virtual:ti,ab,kw | 1.177.615 |
| #2 | 'telemedicine'/exp OR 'teleconsultation'/exp OR 'e counseling'/exp OR 'social media'/exp OR 'internet'/exp OR 'social network'/exp | 179.916 |
| #3 | 'mental health':ti,ab,kw OR 'mental disorders':ti,ab,kw OR 'mental problem*':ti,ab,kw OR 'mental disorder':ti,ab,kw OR 'behavior disorders':ti,ab,kw OR 'behavior disorder':ti,ab,kw OR 'psychiatric disease':ti,ab,kw OR 'psychiatric diseases':ti,ab,kw OR 'psychiatric illness':ti,ab,kw OR 'psychiatric illnesses':ti,ab,kw OR 'psychiatric disorder':ti,ab,kw OR 'psychiatric disorders':ti,ab,kw OR 'mood disorder':ti,ab,kw OR 'mood disorders':ti,ab,kw OR 'psychological problem':ti,ab,kw OR 'psychological disturb*':ti,ab,kw OR 'psychological ill*':ti,ab,kw OR 'psychological disease*':ti,ab,kw OR 'emotional problem*':ti,ab,kw OR 'emotional distress':ti,ab,kw OR 'emotional stress*':ti,ab,kw | 354.374 |
| #4 | 'mental health'/exp OR 'mental disease'/exp OR 'stress'/exp OR 'distress syndrome'/exp OR 'behavior disorder'/exp OR 'mood disorder'/exp OR 'emotional stress'/exp | 2.651.178 |
| #5 | 'depression':ti,ab,kw OR 'depressive disorder':ti,ab,kw OR depressi*:ti,ab,kw OR dysphori*:ti,ab,kw OR dysthymi*:ti,ab,kw OR melancholia*:ti,ab,kw OR 'affective disorder':ti,ab,kw OR 'affective disorders':ti,ab,kw OR 'mood disorder':ti,ab,kw OR 'mood disorders':ti,ab,kw | 578.094 |
| #6 | 'depression'/exp | 505.140 |
| #7 | 'anxiety disorders':ti,ab,kw OR anxiet*:ti,ab,kw OR hypervigilance:ti,ab,kw OR nervousness:ti,ab,kw OR agoraphobia:ti,ab,kw OR catastrophiz*:ti,ab,kw OR phobi*:ti,ab,kw OR panic*:ti,ab,kw | 314.554 |
| #8 | 'anxiety disorder'/exp OR 'anxiety'/exp OR 'hypervigilance'/exp OR 'nervousness'/exp OR 'agoraphobia'/exp OR 'catastrophizing'/exp OR 'phobia'/exp OR 'panic'/exp | 445.655 |
| #9 | stress*:ti,ab,kw OR distress*:ti,ab,kw OR trauma*:ti,ab,kw OR 'post traumatic':ti,ab,kw OR posttraumatic:ti,ab,kw OR 'reactive disorder':ti,ab,kw OR 'adjustment disorder':ti,ab,kw OR 'emotional adjustment':ti,ab,kw OR 'general disorder':ti,ab,kw OR 'general adaptation':ti,ab,kw OR 'emotional adaptation':ti,ab,kw OR 'psychological adjustment':ti,ab,kw OR 'psychologic adaptation':ti,ab,kw OR 'psychological adaptation':ti,ab,kw OR 'adaptive behavior':ti,ab,kw | 1.712.735 |
| #10 | 'posttraumatic stress disorder'/exp OR 'adaptation syndrome'/exp OR 'psychological adjustment'/exp | 68.612 |
| #11 | 'meta analysis':ti,ab,kw OR 'meta-analysis':ti,ab,kw OR metanalysis:ti,ab,kw OR metaanalysis:ti,ab,kw OR 'Systematic review':ti,ab,kw OR 'Systematic reviews':ti,ab,kw OR 'meta synthesis':ti,ab,kw OR metasynthesis:ti,ab,kw | 343.492 |
| #12 | 'systematic review'/exp OR 'meta analysis'/exp | 357.936 |
| #13 | #1 OR #2 | 1.233.821 |
| #14 | #3 OR #4 OR #5 OR #6 OR #7 OR #8 OR #9 OR #10 | 4.185.220 |
| #15 | #11 OR #12 | 446.607 |
| #16 | #13 AND #14 AND #15 | 12.595 |
| #17 | #16 AND (2015:py OR 2016:py OR 2017:py OR 2018:py OR 2019:py OR 2020:py) | 9.119 |

SCOPUS

| N° | Search strategy |  |
| --- | --- | --- |
| #1 | TITLE-ABS-KEY ("Telemedicine" or "digital health" or Telehealth or mHealth or "Mobile Health" or eHealth or e-Health or "Electronic health" or emedicine or e-medicine or "Electronic medicine" or "Remote Consultation" or Teleconsultation* or multimedia or apps or facebook* or twitter* or tweet* or mobile* or phone* or "social media" or ios or android or ipad* or iphone* or ipod* or tablet* or computer* or online or web or "personal digital assistant" or chat or eTherap* or gaming or iCBT or "information technolog*" or "instant messag*" or internet* or "social network*" or "social medi*" or teletherap* or virtual) | 7.343.025 |
| #2 | TITLE-ABS-KEY ("Mental Health" or "Mental Disorders" or "Mental problem*" or "Mental Disorder" or "Behavior Disorders" or "Behavior Disorder" or "Psychiatric disease" or "Psychiatric diseases" or "Psychiatric illness" or "Psychiatric illnesses" or "Psychiatric disorder" or "Psychiatric disorders" or "Mood disorder" or "Mood disorders" or "psychological problem" or "psychological disturb*" or "psychological ill*" or "psychological disease*" or "emotional problem*" or "emotional distress" or "emotional stress*") | 649.748 |
| #3 | TITLE-ABS-KEY ("Depression" or "Depressive Disorder" or Depressi* or dysphori* or dysthymi* or melancholia* or "affective disorder" or "affective disorders" or "mood disorder" or "mood disorders") | 804.063 |
| #4 | TITLE-ABS-KEY (Anxiety Disorders or anxiet* or Hypervigilance or Nervousness or Agoraphobia or Catastrophiz* or Phobi* or Panic*) | 393.369 |
| #5 | TITLE-ABS-KEY (Stress* or Distress* or trauma* or Post-Traumatic or Posttraumatic or “Reactive Disorder” or “Adjustment Disorder” or Emotional Adjustment or "General disorder" or “General Adaptation” or "Emotional Adaptation" or "Psychological Adjustment" or "Psychologic Adaptation" or "Psychological Adaptation" or "Adaptive Behavior") | 70.041 |
| #6 | TITLE-ABS-KEY ("meta analysis" OR "meta-analysis" OR metanalysis OR metaanalysis OR "Systematic review" OR "Systematic reviews" OR "meta synthesis" OR metasynthesis) | 446.574 |
| #7 | #2 OR #3 OR #4 OR #5 | 1.501.942 |
| #8 | #1 AND #6 AND #7 | 6.535 |
| #9 | (#1 AND #6 AND #7) AND ( LIMIT-TO ( PUBYEAR , 2020 ) OR LIMIT-TO ( PUBYEAR , 2019 ) OR LIMIT-TO ( PUBYEAR , 2018 ) OR LIMIT-TO ( PUBYEAR , 2017 ) OR LIMIT-TO ( PUBYEAR , 2016 ) OR LIMIT-TO ( PUBYEAR , 2015 ) ) | 4.600 |

CINAHL Complete (by EBSCOhost)

| N° | Search strategy |  |
| --- | --- | --- |
| S1 | TI (("Telemedicine" or "digital health" or Telehealth or mHealth or "Mobile Health" or eHealth or e-Health or "Electronic health" or emedicine or e-medicine or "Electronic medicine" or "Remote Consultation" or Teleconsultation* or multimedia or apps or facebook* or twitter* or tweet* or mobile* or phone* or "social media" or ios or android or ipad* or iphone* or ipod* or tablet* or computer* or online or web or "personal digital assistant" or chat or eTherap* or gaming or iCBT or "information technolog*" or "instant messag*" or internet* or "social network*" or "social medi*" or teletherap* or virtual)) | 124.530 |
| S2 | AB (("Telemedicine" or "digital health" or Telehealth or mHealth or "Mobile Health" or eHealth or e-Health or "Electronic health" or emedicine or e-medicine or "Electronic medicine" or "Remote Consultation" or Teleconsultation* or multimedia or apps or facebook* or twitter* or tweet* or mobile* or phone* or "social media" or ios or android or ipad* or iphone* or ipod* or tablet* or computer* or online or web or "personal digital assistant" or chat or eTherap* or gaming or iCBT or "information technolog*" or "instant messag*" or internet* or "social network*" or "social medi*" or teletherap* or virtual )) | 234.216 |
| S3 | (MH "Telemedicine") OR (MH "Remote Consultation") OR (MH "Distance Counseling") | 13.303 |
| S4 | S1 OR S2 OR S3 | 305.415 |
| S5 | TI(("Mental Health" or "Mental disorder" or "Mental problem*" or "Mental disorders" or "Behavior Disorders" or "Behavior Disorder" or "Psychiatric disease" or "Psychiatric diseases" or "Psychiatric illness" or "Psychiatric illnesses" or "Psychiatric disorder" or "Psychiatric disorders" or "Mood disorder" or "Mood disorders" or “psychological problem*” or “psychological distress” or “psychological stress*” or “psychological disturb*” or “psychological ill*” or “psychological disease*” or “emotional problem*” or “emotional distress” or “emotional stress*”)) | 57.938 |
| S6 | AB(("Mental Health" or "Mental disorder" or "Mental problem*" or "Mental disorders" or "Behavior Disorders" or "Behavior Disorder" or "Psychiatric disease" or "Psychiatric diseases" or "Psychiatric illness" or "Psychiatric illnesses" or "Psychiatric disorder" or "Psychiatric disorders" or "Mood disorder" or "Mood disorders" or “psychological problem*” or “psychological distress” or “psychological stress*” or “psychological disturb*” or “psychological ill*” or “psychological disease*” or “emotional problem*” or “emotional distress” or “emotional stress*”)) | 24.408 |
| S7 | (MH "Mental Health") OR (MH "Mental Disorders") | 91.256 |
| S8 | TI ((Depressi* or dysphori* or dysthymi* or melancholia* or "affective disorder" or "affective disorders" or "mood disorder" or "mood disorders")) | 62.052 |
| S9 | AB ((Depressi* or dysphori* or dysthymi* or melancholia* or "affective disorder" or "affective disorders" or "mood disorder" or "mood disorders")) | 124.335 |
| S10 | (MH "Depression") OR (MH "Depressive Disorder") | 107.630 |
| S11 | TI ((anxiet* or Hypervigilance or Nervousness or Agoraphobia* or Catastrophiz* or Phobi* or Panic*)) | 26.451 |
| S12 | AB ((anxiet* or Hypervigilance or Nervousness or Agoraphobia* or Catastrophiz* or Phobi* or Panic*)) | 75.508 |
| S13 | (MH "Anxiety Disorders") OR (MH "Anxiety") OR (MH "Panic") | 53.075 |
| S14 | TI ((Stress* or Distress* or trauma* or Post-Traumatic or Posttraumatic or “Reactive Disorder” or “Adjustment Disorder” or Emotional Adjustment or "General disorder" or “General Adaptation” or "Emotional Adaptation" or "Psychological Adjustment" or "Psychologic Adaptation" or "Psychological Adaptation" or "Adaptive Behavior")) | 125.023 |
| S15 | AB ((Stress* or Distress* or trauma* or Post-Traumatic or Posttraumatic or “Reactive Disorder” or “Adjustment Disorder” or Emotional Adjustment or "General disorder" or “General Adaptation” or "Emotional Adaptation" or "Psychological Adjustment" or "Psychologic Adaptation" or "Psychological Adaptation" or "Adaptive Behavior")) | 258.106 |
| S16 | (MH "Trauma and Stressor Related Disorders") OR (MH "Stress Disorders, Traumatic") OR (MH "Stress, Psychological") OR (MH "Adaptation, Psychological") OR (MH "General Adaptation Syndrome") OR (MH "Emotional Adjustment") | 75.986 |
| S17 | S5 OR S6 OR S7 OR S8 OR S9 OR S10 OR S11 OR S12 OR S13 OR S14 OR S15 OR S16 | 639.660 |
| S18 | TI(("meta analysis" OR "meta-analysis" OR metanalysis OR metaanalysis OR "Systematic review" OR "Systematic reviews" OR "meta synthesis" OR metasynthesis)) | 95.097 |
| S19 | AB(("meta analysis" OR "meta-analysis" OR metanalysis OR metaanalysis OR "Systematic review" OR "Systematic reviews" OR "meta synthesis" OR metasynthesis)) | 83.980 |
| S20 | (MH "meta-analysis as topic") OR (MH "review literature as topic") | 827 |
| S21 | S18 OR S19 OR S20 | 127.033 |
| S22 | S4 AND S17 AND S21 | 2.745 |
| S23 | S4 AND S17 AND S21  Published Date: 20150101-20201231 | 2.033 |

Web of Science databases, including Science Citation Index Expanded, Social Sciences Citation Index, Arts & Humanities Citation Index (A&HCI), Emerging Sources Citation Index (ESCI) --2015-presente and Conference Proceedings Citation Index (Clarivate Analytics)

| N° | Search strategy |  |
| --- | --- | --- |
| #1 | TS=("Telemedicine" or "digital health" or Telehealth or mHealth or "Mobile Health" or eHealth or e-Health or "Electronic health" or emedicine or e-medicine or "Electronic medicine" or "Remote Consultation" or Teleconsultation* or multimedia or apps or facebook* or twitter* or tweet* or mobile* or phone* or "social media" or ios or android or ipad* or iphone* or ipod* or tablet* or computer* or online or web or "personal digital assistant" or chat or eTherap* or gaming or iCBT or "information technolog*" or "instant messag*" or internet* or "social network*" or "social medi*" or teletherap* or virtual) | 2.972.495 |
| #2 | TS=("Mental Health" or "Mental Disorders" or "Mental problem*" or "Mental Disorder" or "Behavior Disorders" or "Behavior Disorder" or "Psychiatric disease" or "Psychiatric diseases" or "Psychiatric illness" or "Psychiatric illnesses" or "Psychiatric disorder" or "Psychiatric disorders" or "Mood disorder" or "Mood disorders" or "psychological problem" or "psychological disturb*" or "psychological ill*" or "psychological disease*" or "emotional problem*" or "emotional distress" or "emotional stress*") | 349.898 |
| #3 | TS=("Depression" or "Depressive Disorder" or Depressi* or dysphori* or dysthymi* or melancholia* or "affective disorder" or "affective disorders" or "mood disorder" or "mood disorders") | 588.921 |
| #4 | TS=(Anxiety Disorders or anxiet* or Hypervigilance or Nervousness or Agoraphobia or Catastrophiz* or Phobi* or Panic*) | 323.885 |
| #5 | TS=(Stress* or Distress* or trauma* or (obsessi* NEAR/2 compulsi*) or (obsessive-compulsive) or OCD or (posttraumatic near/1 stress*) or (post-traumatic near/1 stress*) or PTSD or “Reactive Disorder” or “Adjustment Disorder” or “Emotional Adjustment” or "General disorder" or “General Adaptation” or "Emotional Adaptation" or "Psychological Adjustment" or "Psychologic Adaptation" or "Psychological Adaptation" or "Adaptive Behavior") | 2.540.503 |
| #6 | TS=("Systematic Review" or "meta-analysis" or meta-anal* or metaanal* or "systematic review" or "systematic reviews" or "Systematic review" or meta-analys* or metaanalys* or meta-analys* or metaanalis* or metasynthesis or meta-synthesis or “meta synthesis”) | 479.059 |
| #7 | #2 or #3 or #4 or #5 | 3.293.733 |
| #8 | #1 and #6 and #7 | 10.023 |
| #9 | #1 and #6 and #7  Refined by: PUBLICATION YEARS: ( 2020 OR 2019 OR 2018 OR 2017 OR 2016 OR 2015 ) | 7.703 |
