# Supplementary material for: Development of a Framework for the Implementation of Synchronous Digital Mental Health: Realist Synthesis of Systematic Reviews
Source: JMIR Ment Health. 2022 Mar 29;9(3):e34760. doi: 10.2196/34760 (PMC9006141; doi:10.2196/34760)
Supplement: Multimedia Appendix 3 [file mental_v9i3e34760_app3.docx]

**Articles that passed full-text review and were excluded.**

| **Article** | **Exclusion Reason** |
| --- | --- |
| Economic evaluation and costs of telepsychiatry programmes: A systematic review | Interventions that lack a synchronic component |
| COVID-19 and Telepsychiatry: Development of Evidence-Based Guidance for Clinicians | Not a systematic review |
| Barriers to Use of Telepsychiatry: Clinicians as Gatekeepers | Not a systematic review |
| The Alliance-Outcome Relation in Internet-Based Interventions for Psychological Disorders: A Correlational Meta-Analysis | Not a systematic review |
| Therapist-supported internet-based cognitive behavior therapy for stress, anxiety, and depressive symptoms among postpartum women: A systematic review and meta-analysis | Interventions that lack a synchronic component |
| Effectiveness and Safety of Using Chatbots to Improve Mental Health: Systematic Review and Meta-Analysis | Interventions that lack a synchronic component |
| Effect of cognitive behavioral therapy on anxiety and depression of infertile women: A meta-analysis | Not on the subject of e-health |
| Developing a General Extended Technology Acceptance Model for E-Learning (GETAMEL) by analysing commonly used external factors | Not on the subject of e-health |
| 6 Internet-supported versus face-to-face cognitive behavior therapy for depression | Interventions that lack a synchronic component |
| Computer- or web-based interventions for perinatal mental health: A systematic review | Other target population |
| Behavioral Interventions Targeting Chronic Pain, Depression, and Substance Use Disorder in Primary Care | Not on the subject of e-health |
| Current Status and Future Directions of mHealth Interventions for Health System Strengthening in India: Systematic Review | Other target population |
| eHealth to Redress Psychotherapy Access Barriers Both New and Old: A Review of Reviews and Meta-Analyses | Not a systematic review |
| Barriers and Facilitation Measures Related to People With Mental Disorders When Using the Web: A Systematic Review | Interventions that lack a synchronic component |
| The effect of e-mental health interventions on academic performance in university and college students: A meta-analysis of randomized controlled trials | Interventions that lack a synchronic component |
| Effectiveness of Web-Delivered Acceptance and Commitment Therapy in Relation to Mental Health and Well-Being: A Systematic Review and Meta-Analysis | Interventions that lack a synchronic component |
| Effects of nonpharmacological interventions on depressive symptoms and depression among nursing students: A systematic review and meta-analysis | Not on the subject of e-health |
| The Effectiveness of Mindfulness- and Acceptance-Based Interventions for Informal Caregivers of People With Dementia: A Meta-Analysis | Not on the subject of e-health |
| Cognitive Behavioural Therapy for Insomnia (CBT-I) to treat depression: A systematic review | Interventions that lack a synchronic component |
| Comparison of treatment options for depression in heart failure: A network meta-analysis | Not on the subject of e-health |
| Cost-Utility and Cost-Effectiveness Studies of Telemedicine, Electronic, and Mobile Health Systems in the Literature: A Systematic Review | Other target population |
| eHealth interventions for the prevention of depression and anxiety in the general population: a systematic review and meta-analysis | Other target population / Interventions that lack a synchronic component |
| Collaborative Care for Psychiatric Disorders in Older Adults: A Systematic Review | Interventions that lack a synchronic component |
| Effectiveness of psychotherapy compared to pharmacotherapy for the treatment of anxiety and depressive disorders in adults: a literature review | Not a systematic review / Not on the subject of e-health |
| The Clinical Effectiveness of Cognitive Behavioral Therapy for Patients with Insomnia and Depression: A Systematic Review and Meta-Analysis | Interventions that lack a synchronic component |
| Can smartphone mental health interventions reduce symptoms of anxiety? A meta-analysis of randomized controlled trials | Interventions that lack a synchronic component |
| Digital Peer Support Mental Health Interventions for People With a Lived Experience of a Serious Mental Illness: Systematic Review | Other target population |
| Early psychological interventions for posttraumatic stress, depression and anxiety after traumatic injury: A systematic review and meta-analysis | Not on the subject of e-health |
| Effects of Internet-Based Psychological Interventions on Emotional Distress and Quality of Life in Adult Cancer Patients: Systematic Review | Interventions that lack a synchronic component |
| Cybertherapy: a scientific model? A text mining analysis of published abstracts | Not a systematic review |
| The Effect of Technology-Based Interventions on Pain, Depression, and Quality of Life in Patients With Cancer: A Systematic Review of Randomized Controlled Trials | Other target population |
| The Benefit of Web- and Computer-Based Interventions for Stress: A Systematic Review and Meta-Analysis | Interventions that lack a synchronic component |
| The Efficacy of Multi-component Positive Psychology Interventions: A Systematic Review and Meta-analysis of Randomized Controlled Trials | Not on the subject of e-health |
| Clinician behaviors in telehealth care delivery: a systematic review | Other target population |
| Considering the Therapeutic Alliance in Digital Mental Health Interventions | Unable to retrieve |
| Application of Synchronous Text-Based Dialogue Systems in Mental Health Interventions: Systematic Review | Other target population |
| The effect of telepsychiatric modalities on reduction of readmissions in psychiatric settings: A systematic review | Other target population / Interventions that lack a synchronic component |
| Effect of psychotherapy for depression on quality of life: meta-analysis | Not on the subject of e-health / Interventions that lack a synchronic component |
| Effectiveness of Cognitive Behavioral Therapy for Caregivers of People with Dementia: A Systematic Review and Meta-Analysis | Interventions that lack a synchronic component |
| The Effect of Telehealth Interventions on Quality of Life of Cancer Patients: A Systematic Review and Meta-Analysis | Other target population |
| Digital Mental Health Interventions for Depression, Anxiety, and Enhancement of Psychological Well-Being Among College Students: Systematic Review | Interventions that lack a synchronic component |
| Application and Effectiveness of Telehealth to Support Severe Mental Illness Management: Systematic Review | Other target population |
| Efficacy of Experiential Dynamic Therapy for Psychiatric Conditions: A Meta-Analysis of Randomized Controlled Trials | Not on the subject of e-health |
| The efficacy of app-supported smartphone interventions for mental health problems: a meta-analysis of randomized controlled trials | Interventions that lack a synchronic component |
| Attrition and Adherence in Smartphone-Delivered Interventions for Mental Health Problems: A Systematic and Meta-Analytic Review | Interventions that lack a synchronic component |
| The effectiveness and cost-effectiveness of e-health interventions for depression and anxiety in primary care: A systematic review and meta-analysis | Interventions that lack a synchronic component |
| Computerised Cognitive Behavioural Therapy for Psychological Distress in Patients with Physical Illnesses: A Systematic Review | Interventions that lack a synchronic component |
| The effect of telehealth versus usual care for home-care patients with long-term conditions: A systematic review, meta-analysis and qualitative synthesis | Other target population |
| Do adjuvant interventions improve treatment outcome in adult patients with posttraumatic stress disorder receiving trauma-focused psychotherapy? A systematic review | Not on the subject of e-health |
| Computerized cognitive training and functional recovery in major depressive disorder: A meta-analysis | Interventions that lack a synchronic component / Not on the subject of e-health |
| Delivery of cognitive behavioural therapy to workers: a systematic review | Interventions that lack a synchronic component |
| The efficacy of cognitive behavioral therapy for Chinese people: A meta-analysis | Interventions that lack a synchronic component |
| The Efficacy and Acceptability of Third-Wave Behavioral and Cognitive eHealth Treatments: A Systematic Review and Meta-Analysis of Randomized Controlled Trials | Interventions that lack a synchronic component |
| Are claims of non-inferiority of Internet and computer-based cognitive-behavioural therapy compared with in-person cognitive-behavioural therapy for adults with anxiety disorders supported by the evidence from head-to-head randomised controlled trials? A systematic review | Interventions that lack a synchronic component |
| Behavioural activation for depression in older people: systematic review and meta-analysis | Interventions that lack a synchronic component |
| The Effectiveness of Prompts to Promote Engagement With Digital Interventions: A Systematic Review | Interventions that lack a synchronic component |
| Digital Characteristics and Dissemination Indicators to Optimize Delivery of Internet-Supported Mindfulness-Based Interventions for People With a Chronic Condition: Systematic Review | Other target population |
| Effectiveness of Internet-Based Interventions for the Prevention of Mental Disorders: A Systematic Review and Meta-Analysis | Interventions that lack a synchronic component |
| Efficacy of Telepsychiatry in Refugee Populations: A Systematic Review of the Evidence | Other target population |
| Designing and Using Digital Mental Health Interventions for Older Adults: Being Aware of Digital Inequality | Not a systematic review |
| The effect of cognitive-behavioral therapy on psychological distress in the mothers of preterm infants: a systematic review and meta-analysis | Not on the subject of e-health |
| Effectiveness of text messaging interventions for the management of depression: A systematic review and meta-analysis | Interventions that lack a synchronic component / Other target population |
| EFFECTIVENESS OF INTERNET-DELIVERED COGNITIVE BEHAVIORAL THERAPY FOR POSTTRAUMATIC STRESS DISORDER: A SYSTEMATIC REVIEW AND META-ANALYSIS | Interventions that lack a synchronic component |
| The effectiveness of mHealth for self-management in improving pain, psychological distress, fatigue, and sleep in cancer survivors: a systematic review | Interventions that lack a synchronic component / Other target population |
| Acceptability of internet-based cognitive behavioural therapy (i-CBT) for post-traumatic stress disorder (PTSD): a systematic review | Interventions that lack a synchronic component |
| EFFECTS OF TELEHEALTH BY ALLIED HEALTH PROFESSIONALS AND NURSES IN RURAL AND REMOTE AREAS: A SYSTEMATIC REVIEW AND META-ANALYSIS | Other target population |
| Effectiveness of online mindfulness-based interventions in improving mental health: A review and meta-analysis of randomised controlled trials | Other target population |
| Effectiveness of eHealth interventions for reducing mental health conditions in employees: A systematic review and meta-analysis | Interventions that lack a synchronic component |
| Efficacy of cognitive behavioural therapy delivered over the Internet for depressive symptoms: A systematic review and meta-analysis | Interventions that lack a synchronic component |
| THE EFFICACY OF INTERNET-BASED INTERVENTIONS FOR STRESS MANAGEMENT: A META-ANALYSIS | Not a systematic review |
| Acceptance and Commitment Therapy as a Treatment for Anxiety and Depression: A Review | Not on the subject of e-health / Not a systematic review |
| Effectiveness of a freely available computerised cognitive behavioural therapy programme (MoodGYM) for depression: Meta-analysis | Interventions that lack a synchronic component |
| Effectiveness of cognitive behavioural therapy for anxiety and depression in primary care: a meta-analysis | Interventions that lack a synchronic component |
| Effectiveness of training methods for delivery of evidence-based psychotherapies: a systematic review | Interventions that lack a synchronic component |
| Computerized Cognitive Behavior Therapy for Anxiety and Depression in Rural Areas: A Systematic Review | Not a systematic review |
| The Application of Positive Psychotherapy in Mental Health Care: A Systematic Review | Not on the subject of e-health |
| Effects of Internet-based psycho-educational interventions on mental health and quality of life among cancer patients: a systematic review and meta-analysis | Other target population |
| Are Trials of Computerized Therapy Generalizable? A Multidimensional Meta-analysis | Interventions that lack a synchronic component |
| Computer-Assisted Cognitive-Behavior Therapy for Depression: A Systematic Review and Meta-Analysis | Interventions that lack a synchronic component |
| Effectiveness of mindfulness-based stress reduction and mindfulness-based cognitive therapies on people living with HIV: A systematic review and meta-analysis | Not on the subject of e-health |
| Efficacy of online lifestyle interventions targeting lifestyle behaviour change in depressed populations: A systematic review | Interventions that lack a synchronic component |
| Behavioural Activation for Depression in Informal Caregivers: A Systematic Review and Meta-Analysis of Randomised Controlled Clinical Trials | Interventions that lack a synchronic component / Not on the subject of e-health |
| Cognitive-behavioral therapy for treatment-resistant depression in adults and adolescents: a systematic review | Not on the subject of e-health |
| Effects of mindfulness-based interventions on quality of life of women with breast cancer: a systematic review | Not on the subject of e-health |
| Effects of mindfulness-based stress reduction on anxiety symptoms in young people: A systematic review and meta-analysis | Not on the subject of e-health |
| Internet Interventions for Adults with Anxiety and Mood Disorders: A Narrative Umbrella Review of Recent Meta-Analyses | Systematic review not composed of primary studies |
| Mental Health Smartphone Apps: Review and Evidence-Based Recommendations for Future Developments | Not a systematic review |
| Gamification and Adherence to Web-Based Mental Health Interventions: A Systematic Review | Interventions that lack a synchronic component |
| Online health research and health anxiety: A systematic review and conceptual integration | Not on the subject of e-health |
| Improving Employee Well-Being and Effectiveness: Systematic Review and Meta-Analysis of Web-Based Psychological Interventions Delivered in the Workplace | Other target population |
| Gamification in Apps and Technologies for Improving Mental Health and Well-Being: Systematic Review | Interventions that lack a synchronic component |
| Meta-Analysis of the Efficacy of Virtual Reality Exposure Therapy for Social Anxiety | Interventions that lack a synchronic component |
| Internet and mobile interventions for depression: Opportunities and challenges | Not a systematic review |
| A Mobile Phone App to Improve the Mental Health of Taxi Drivers: Single-Arm Feasibility Trial | Not a systematic review |
| An Internet-Based Intervention (Mamma Mia) for Postpartum Depression: Mapping the Development from Theory to Practice | Not a systematic review |
| Internet and Computer-Based Cognitive Behavioral Therapy for Anxiety and Depression in Youth: A Meta-Analysis of Randomized Controlled Outcome Trials | Other target population |
| Internet-Delivered Early Interventions for Individuals Exposed to Traumatic Events: Systematic Review | Interventions that lack a synchronic component |
| Evaluation Methods for Assessing Users' Psychological Experiences of Web-Based Psychosocial Interventions: A Systematic Review | Interventions that lack a synchronic component |
| Evidence for telehealth group-based treatment: A systematic review | Other target population |
| Geriatric Telepsychiatry: Systematic Review and Policy Considerations | Other target population |
| Mental Health Mobile Apps for Preadolescents and Adolescents: A Systematic Review | Other target population |
| Internet interventions for mental health in university students: A systematic review and meta-analysis | Interventions that lack a synchronic component |
| Internet- and mobile-based aftercare and relapse prevention in mental disorders: A systematic review and recommendations for future research | Interventions that lack a synchronic component |
| Guided Internet-based cognitive behavioral therapy for mild and moderate depression: A benchmarking study | Not a systematic review |
| Internet-Delivered Acceptance and Commitment Therapy for Anxiety Treatment: Systematic Review | Interventions that lack a synchronic component |
| The Generalizability of Randomized Controlled Trials of Self-Guided Internet-Based Cognitive Behavioral Therapy for Depressive Symptoms: Systematic Review and Meta-Regression Analysis | Interventions that lack a synchronic component |
| Establishing and Governing e-Mental Health Care in Australia: A Systematic Review of Challenges and A Call For Policy-Focussed Research | Other target population |
| Mobile Apps for Health Behavior Change in Physical Activity, Diet, Drug and Alcohol Use, and Mental Health: Systematic Review | Other target population |
| Mental Health-Related Digital Use by University Students: A Systematic Review | Other target population |
| Internet-Delivered Cognitive Behavioral Therapy for Anxiety Disorders in Open Community Versus Clinical Service Recruitment: Meta-Analysis | Interventions that lack a synchronic component |
| Mindfulness-based Online Interventions for Mental Health Treatment: A Systematic Review and Meta-analysis | Interventions that lack a synchronic component |
| Internet interventions for depression: new developments | Not a systematic review |
| Internet-Based Interventions for Carers of Individuals With Psychiatric Disorders, Neurological Disorders, or Brain Injuries: Systematic Review | Other target population |
| Online Health Services for the Prevention of Stress-associated Psychological Impairments at the Workplace | Interventions that lack a synchronic component |
| Nonpharmacological Interventions for Depressive Symptoms in End-Stage Renal Disease: A Systematic Review | Not on the subject of e-health |
| Internet-delivered cognitive behavioral therapies for late-life depressive symptoms: a systematic review and meta-analysis | Interventions that lack a synchronic component |
| Internet-based cognitive behavioural therapy for subthreshold depression: a systematic review and meta-analysis | Interventions that lack a synchronic component |
| Telemental health: A status update | Not a systematic review |
| Social anxiety apps: a systematic review and assessment of app descriptors across mobile store platforms | Interventions that lack a synchronic component |
| Response and Remission Rates in Internet-Based Cognitive Behavior Therapy: An Individual Patient Data Meta-Analysis | Not a systematic review |
| Utilization of Mobile Mental Health Services among Syrian Refugees and Other Vulnerable Arab Populations-A Systematic Review | Interventions that lack a synchronic component |
| Telemedicine interventions for medication adherence in mental illness: A systematic review | Other target population |
| There is a non -evidence -based app for that: A systematic review and mixed methods analysis of depression- and anxiety -related apps that incorporate unrecognized techniques | Not a systematic review |
| A Systematic Review of Predictors of, and Reasons for, Adherence to Online Psychological Interventions | Interventions that lack a synchronic component |
| Videoconferencing Psychotherapy and Depression: A Systematic Review | Other target population |
| Videoconferencing psychological therapy and anxiety: a systematic review | Other target population |
| Telepsychology for Posttraumatic Stress Disorder: A systematic review | Interventions that lack a synchronic component |
| Technological State of the Art of Electronic Mental Health Interventions for Major Depressive Disorder: Systematic Literature Review | Interventions that lack a synchronic component |
| Virtual reality exposure therapy for anxiety and related disorders: A meta-analysis of randomized controlled trials | Interventions that lack a synchronic component |
| Web-based interventions for comorbid depression and chronic illness: a systematic review | Interventions that lack a synchronic component |
| Technology-Delivered Psychotherapeutic Interventions in Improving Depressive Symptoms Among People with HIV/AIDS: A Systematic Review and Meta-analysis of Randomised Controlled Trials | Interventions that lack a synchronic component |
| Serious Games for Psychotherapy: A Systematic Review | Interventions that lack a synchronic component |
| Virtual Reality Therapy in Social Anxiety Disorder | Not a systematic review |
| A systematic review of the effectiveness of mobile apps for common mental health disorders: Policy implications and future recommendations | Unable to retrieve |
| A SYSTEMATIC REVIEW OF ONLINE INTERVENTIONS FOR ADDRESSING PSYCHOLOGICAL DISTRESS IN RHEUMATOID ARTHRITIS AND OTHER LONG-TERM CONDITIONS | Not a systematic review |
| Serious game as a therapeutic tool in psychiatry: A systematic review | Other target population |
| Virtual reality in the assessment, understanding, and treatment of mental health disorders | Interventions that lack a synchronic component |
| What Works and What Doesn't Work? A Systematic Review of Digital Mental Health Interventions for Depression and Anxiety in Young People | Other target population |
| Systematic review with meta-analysis: online psychological interventions for mental and physical health outcomes in gastrointestinal disorders including irritable bowel syndrome and inflammatory bowel disease | Other target population |
| Reducing caregiver stress with internet-based interventions: a systematic review of open-label and randomized controlled trials | Publication year before 2015 |
| A systematic review and meta-analysis on the efficacy of Internet-delivered behavioral activation | Interventions that lack a synchronic component |
| A Systematic Review of Cognitive Behavioral Therapy and Behavioral Activation Apps for Depression | Systematic review not composed of primary studies |
| Usability Evaluations of Mobile Mental Health Technologies: Systematic Review | Interventions that lack a synchronic component |
| Virtual Reality and Symptoms Management of Anxiety, Depression, Fatigue, and Pain: A Systematic Review | Other target population |
| Predictors of treatment dropout in self-guided web-based interventions for depression: an 'individual patient data' meta-analysis | Not a systematic review |
| A systematic review of technology-based preoperative preparation interventions for child and parent anxiety | Other target population |
| Update of Recent Literature on Remotely Delivered Psychotherapy Interventions for Anxiety and Depression | Systematic review not composed of primary studies |
| Serious Games for Mental Health: Are They Accessible, Feasible, and effective? A Systematic Review and Meta-analysis | Interventions that lack a synchronic component |
| Technology-Supported Interventions for Pregnant Women A Systematic Review | Interventions that lack a synchronic component |
| Use of Technology-Based Tools to Support Adolescents and Young Adults With Chronic Disease: Systematic Review and Meta-Analysis | Other target population |
| A Systematic Review of Electronic Mindfulness-Based Therapeutic Interventions for Weight, Weight-Related Behaviors, and Psychological Stress | Interventions that lack a synchronic component |
| Smartphone Apps for the Treatment of Mental Disorders: Systematic Review | Interventions that lack a synchronic component |
| Telehealth and eHealth interventions for posttraumatic stress disorder | Not a systematic review |
| Systematic review and meta-analysis of transdiagnostic psychological treatments for anxiety and depressive disorders in adulthood | Not on the subject of e-health |
| User Engagement in Mental Health Apps: A Review of Measurement, Reporting, and Validity | Other target population |
| Virtual Reality Applications for Stress Management Training in the Military | Interventions that lack a synchronic component |
| Web-Based Tools and Mobile Applications To Mitigate Burnout, Depression, and Suicidality Among Healthcare Students and Professionals: a Systematic Review | Systematic review not composed of primary studies |
| Systematic review of technology-based interventions to improve anxiety, depression, and health-related quality of life among patients with prostate cancer | Interventions that lack a synchronic component |
| The Use of Mobile Apps and SMS Messaging as Physical and Mental Health Interventions: Systematic Review | It's a protocol |
| The Use of Social Networking Sites in Mental Health Interventions for Young People: Systematic Review | Other target population |
| Treatment of Depression in Primary Care with Computerized Psychological Therapies: Systematic Reviews | Systematic review not composed of primary studies |
| Use of Social Robots in Mental Health and Well-Being Research: Systematic Review | Interventions that lack a synchronic component |
| Web-Based Mindfulness Interventions for Mental Health Treatment: Systematic Review and Meta-Analysis | Interventions that lack a synchronic component |
| A Systematic Review and Meta-Analysis of e-Mental Health Interventions to Treat Symptoms of Posttraumatic Stress | Interventions that lack a synchronic component |
| A review of web-based technology in behavioural activation | Not a systematic review |
| A Review and Meta-Analysis of Perfectionism Interventions: Comparing Face-to-Face With Online Modalities | Not on the subject of e-health |
| Web-Based Mindfulness Interventions for People With Physical Health Conditions: Systematic Review | Interventions that lack a synchronic component |
| Virtual reality in the psychological treatment for mental health problems: An systematic review of recent evidence | Other target population |
| A systematic review of the effectiveness of mobile apps for monitoring and management of mental health symptoms or disorders | Interventions that lack a synchronic component |
| A Systematic Review and Meta-analysis of Telephone-Based Therapy Targeting Depressive Symptoms Among Low-Income People Living with HIV | Interventions that lack a synchronic component |
| Virtual Reality Exercise for Anxiety and Depression: A Preliminary Review of Current Research in an Emerging Field | Interventions that lack a synchronic component |
| Systematic review of the information and communication technology features of web- and mobile-based psychoeducational interventions for depression | Other target population |
| Computerised therapies for anxiety and depression in children and young people: A systematic review and meta-analysis | Other target population |
| Cognitive behaviour therapy for depression in primary care: systematic review and meta-analysis | Not on the subject of e-health |
| Effectiveness of pharmacological or psychological interventions for smoking cessation in smokers with major depression or depressive symptoms: A systematic review of the literature | Not on the subject of e-health |
| Data Mining Algorithms and Techniques in Mental Health: A Systematic Review | Interventions that lack a synchronic component / Not on the subject of e-health |
| Internet-Delivered Cognitive Behavioural Therapy for Major Depressive Disorder and Anxiety Disorders: Recommendations [Internet] | Not a systematic review |
| The Effectiveness Of Cognitive Behaviour Therapy (CBT) And Internet Cognitive Behavior Therapy (ICBT) As Non-Pharmacological Interventions On Patients With Anxiety: A Systematic Review | Interventions that lack a synchronic component |
| Cognitive behavior therapy for health anxiety: systematic review and meta-analysis of clinical efficacy and health economic outcomes | Interventions that lack a synchronic component |
| The effectiveness of behavioural and cognitive behavioural therapies for insomnia on depressive and fatigue symptoms: A systematic review and network meta-analysis | Interventions that lack a synchronic component / Not on the subject of e-health |
| Efficacy of treatments for anxiety disorders: A meta-analysis | Interventions that lack a synchronic component |
| Effectiveness of psycho-educational interventions with telecommunication technologies on emotional distress and quality of life of adult cancer patients: a systematic review | Other target population |
| Computer therapy for the anxiety and depression disorders is effective, acceptable and practical healthcare: an updated meta-analysis | Interventions that lack a synchronic component |
| Digital health technology for use in patients with serious mental illness: a systematic review of the literature | Interventions that lack a synchronic component |
| Barriers and facilitators to the integration of web-based interventions into routine care | Not a systematic review |
| Effectiveness of psychosocial interventions on stroke survivors, their carers and stroke-carer dyads | Not a systematic review |
| The effects of meditation, yoga, and mindfulness on depression, anxiety, and stress in tertiary education students: A meta-analysis | Not on the subject of e-health |
| Cost-utility analyses of cognitive-behavioural therapy of depression: A systematic review | Interventions that lack a synchronic component / Not on the subject of e-health |
| Effects of yoga on depressive symptoms in people with mental disorders: A systematic review and meta-analysis | Not on the subject of e-health |
| Efficacy of intensive short-term dynamic psychotherapy in mood disorders: A critical review | Not on the subject of e-health |
| Characteristics and effectiveness of cognitive behavioral therapy for older adults living in residential care: a systematic review | Not on the subject of e-health |
| Effectiveness of the mHealth technology in improvement of healthy behaviors in an elderly population-a systematic review | Other target population |
| The effect of nurse-led telephone follow-up on anxiety in cancer patients: A systematic review | Not a systematic review |
| Effectiveness of Psychological and Educational Interventions to Prevent Depression in Primary Care: A Systematic Review and Meta-Analysis | Interventions that lack a synchronic component / Not on the subject of e-health |
| Acceptance and Commitment Therapy in group format for anxiety and depression. A systematic review | Not on the subject of e-health |
| Effectiveness and Acceptability of Low-intensity Psychological Interventions on the Well-being of Older Adults: A Systematic Review | Interventions that lack a synchronic component / Not on the subject of e-health |
| Computer-delivered and web-based interventions to improve depression, anxiety, and psychological well-being of university students: a systematic review and meta-analysis | Interventions that lack a synchronic component |
| A comparison of VRE and CBT in combat veterans diagnosed with PTSD: A meta-analysis | Not a systematic review / Not on the subject of e-health |
| Economic evaluations of Internet interventions for mental health: a systematic review | Interventions that lack a synchronic component |
| Are acceptance and mindfulness‐based interventions 'value for money'? Evidence from a systematic literature review | Interventions that lack a synchronic component |
| Blending face-to-face and internet-based interventions for the treatment of mental disorders in adults: Systematic review | Interventions that lack a synchronic component |
| Effects of Internet-Based Cognitive Behavioral Therapy in Routine Care for Adults in Treatment for Depression and Anxiety: Systematic Review and Meta-Analysis | Interventions that lack a synchronic component |
| Effectiveness of digital psychological interventions for mental health problems in low-income and middle-income countries: a systematic review and meta-analysis | Interventions that lack a synchronic component / Other target population |
| Efficacy and acceptability of mindfulness-based interventions for military veterans: A systematic review and meta-analysis | Not on the subject of e-health |
| Telehealth Services Designed for Women: An Evidence Map [Internet] | Not a systematic review |
| Components and Outcomes of Internet-Based Interventions for Caregivers of Older Adults: Systematic Review | Interventions that lack a synchronic component |
| Efficacy of Cognitive Behavioral Therapy for Generalized Anxiety Disorder in Older Adults: Systematic Review, Meta-Analysis, and Meta-Regression | Interventions that lack a synchronic component / Not on the subject of e-health |
| Cognitive behavioural therapy for the treatment of depression in people with multiple sclerosis: a systematic review and meta-analysis | Other target population / Not on the subject of e-health |
| Effect of cognitive-behavioral therapy for anxiety disorders on quality of life: a meta-analysis | Interventions that lack a synchronic component / Not on the subject of e-health |
| Effects of preventive online mindfulness interventions on stress and mindfulness: A meta-analysis of randomized controlled trials | Interventions that lack a synchronic component / Other target population |
| Effectiveness of cognitive behavior therapy on posttraumatic growth in patients after breast cancer surgery:a Meta-analysis | Unable to retrieve |
| Effect of psycho-educational interventions on quality of life in patients with implantable cardioverter defibrillators: A meta-analysis of randomized controlled trials | Not on the subject of e-health |
| Do guided internet-based interventions result in clinically relevant changes for patients with depression? An individual participant data meta-analysis | Interventions that lack a synchronic component |
| effectiveness of interventions for managing multiple high-burden chronic diseases in older adults: A systematic review and meta-analysis | Interventions that lack a synchronic component |
| Do online mental health services improve help-seeking for young people? A systematic review | Other target population |
| Efficacy of psychosocial interventions on psychological outcomes among people with cardiovascular diseases: A systematic review and meta-analysis | Not on the subject of e-health |
| Cost effectiveness of guided Internet-based interventions for depression in comparison with control conditions: An individual–participant data meta-analysis | Interventions that lack a synchronic component |
| Computer-based versus in-person interventions for preventing and reducing stress in workers | Interventions that lack a synchronic component |
| Efficacy of mindfulness-based cognitive therapy in prevention of depressive relapse an individual patient data meta-analysis from randomized trials | Not on the subject of e-health |
| Effects of mindfulness meditation on anxiety, depression, stress, and mindfulness in nursing students: A meta-analysis and trial sequential analysis of randomized controlled trials | Not on the subject of e-health |
| Effectiveness of health web-based and mobile app-based interventions designed to improve informal caregiver's well-being and quality of life: A systematic review | Interventions that lack a synchronic component / Other target population |
| Effectiveness of cognitive behavioural therapy for chronic obstructive pulmonary disease patients: A systematic review and meta‐analysis | Interventions that lack a synchronic component |
| Does CBT have lasting effects in the treatment of PTSD after one year of follow-up? A systematic review of randomized controlled trials | Not on the subject of e-health |
| Effectiveness of computer-mediated interventions for informal carers of people with dementia-a systematic review | Interventions that lack a synchronic component |
| Efficacy of deprexis, an internet-based depression treatment: A meta-analysis of ten randomized controlled trials | Interventions that lack a synchronic component / Not a systematic review |
| Effectiveness of mindfulness-and relaxation-based eHealth interventions for patients with medical conditions: A systematic review | Interventions that lack a synchronic component |
| Electronic and mobile health applications improve emotional functioning: A systematic review and meta-analysis of unguided electronic and mobile health technologies for patients with chronic pain | Not a systematic review |
| Effectiveness of Psychological and/or Educational Interventions in the Prevention of Anxiety: A Systematic Review, Meta-analysis, and Meta-regression | Not on the subject of e-health |
| Collateral outcomes in e-mental health: a systematic review of the evidence for added benefits of computerized cognitive behavior therapy interventions for mental health | Interventions that lack a synchronic component / Other target population |
| Efficacy and acceptability of group cognitive behavioral therapy for depression: a systematic review and meta-analysis | Not on the subject of e-health |
| Cost-effectiveness of interventions for treating anxiety disorders: A systematic review | Interventions that lack a synchronic component |
| Economic evaluations of internet- and mobile-based interventions for the treatment and prevention of depression: A systematic review | Interventions that lack a synchronic component |
| Efficacy of music therapy interventions in the treatment of mood disorders: A systematic review | Not a systematic review |
| Comparative efficacy of dyadic interventions over individual interventions for treating depression | Not a systematic review |
| The acceptability and usability of digital health interventions for adults with depression, anxiety, and somatoform disorders: Qualitative systematic review and meta-synthesis | Interventions that lack a synchronic component |
| The Effect of Psychotherapy on Quality of Life in IBD Patients: A Systematic Review | Not on the subject of e-health |
| EFFECTIVENESS OF COMMUNITY-BASED INTERVENTIONS FOR THE PREVENTION AND TREATMENT OF MENTAL HEALTH PROBLEMS AND MENTAL DISORDERS IN THE GENERAL POPULATION: SYSTEMATIC REVIEW | Not a systematic review |
| Effects of programmed exercise on depressive symptoms in midlife and older women: A meta-analysis of randomized controlled trials | Not on the subject of e-health |
| Effectiveness of occupational e-mental health interventions: A systematic review and meta-analysis of randomized controlled trials | Interventions that lack a synchronic component |
| Efficacy and Moderators of Internet-Based Interventions in Adults with Subthreshold Depression: An Individual Participant Data Meta-Analysis of Randomized Controlled Trials | Interventions that lack a synchronic component |
| Effectiveness of mindfulness meditation in intervention for anxiety: A meta-analysis | Unable to retrieve |
| The efficacy of internet-delivered treatment for generalized anxiety disorder: A systematic review and meta-analysis | Interventions that lack a synchronic component |
| Digital Interventions for Screening and Treating Common Mental Disorders or Symptoms of Common Mental Illness in Adults: Systematic Review and Meta-analysis | Interventions that lack a synchronic component |
| Cognitive behavioral therapy online for adult depression: A 10 year systematic literature review | Interventions that lack a synchronic component |
| Are Digitally Delivered Psychological Interventions for Depression the Way Forward? A Review | Interventions that lack a synchronic component |
| Digitally Delivered Psychological Interventions for Anxiety Disorders: a Comprehensive Review | Interventions that lack a synchronic component |
| Effectiveness of a tailored, integrative Internet intervention (deprexis) for depression: updated meta-analysis | Interventions that lack a synchronic component |
| Comparison of pharmacological versus psychological therapies for management of anxiety in COPD patients | Not a systematic review |
| Adherence to Internet-based and face-to-face cognitive behavioural therapy for depression: a meta-analysis | Interventions that lack a synchronic component |
| Efficacy of synchronous telepsychology interventions for people with anxiety, depression, posttraumatic stress disorder, and adjustment disorder: A rapid evidence assessment | Not a systematic review |
| The effectiveness of various computer-based interventions for patients with chronic pain or functional somatic syndromes: A systematic review and meta-analysis | Interventions that lack a synchronic component |
| Computer-assisted cognitive-behavior therapy for depression in primary care: Systematic review and meta-analysis | Interventions that lack a synchronic component |
| Effects of cognitive behavioral therapy on anxiety and depression in patients with chronic obstructive pulmonary disease: A Meta-analysis and Systematic Review | Not on the subject of e-health |
| The effects of psychological interventions on depression and anxiety among Chinese adults with cancer: a meta-analysis of randomized controlled studies | Not on the subject of e-health |
| Effectiveness of internet-based interventions for children, youth, and young adults with anxiety and/or depression: a systematic review and meta-analysis | Other target population |
| Cognitive behavioral therapy for primary care depression and anxiety: a secondary meta-analytic review using robust variance estimation in meta-regression | Interventions that lack a synchronic component |
| Effectiveness of telephone‐based interventions on health‐related quality of life and prognostic outcomes in breast cancer patients and survivors—A meta‐analysis | Other target population |
| Effectiveness of mindfulness-based stress reduction (MBSR) on symptom variables and health-related quality of life in breast cancer patients—a systematic review and meta-analysis | Not on the subject of e-health |
| Effects of cognitive behavioral therapy on anxiety and depression in patients with chronic obstructive pulmonary disease: A meta-analysis and systematic review | Interventions that lack a synchronic component / Not on the subject of e-health |
| Efficacy, patient-doctor relationship, costs and benefits of utilizing telepsychiatry for the management of post-traumatic stress disorder (PTSD): a systematic review | Systematic review not composed of primary studies |
| Mobile applications in mental health: A systematic review of efficacy | Interventions that lack a synchronic component |
| Interformat reliability of digital psychiatric self-report questionnaires: a systematic review | Publication year before 2015 |
| Online, social media and mobile technologies for psychosis treatment: a systematic review on novel user-led interventions | Publication year before 2015 |
| Guided Internet-based vs. face-to-face cognitive behavior therapy for psychiatric and somatic disorders: a systematic review and meta-analysis | Publication year before 2015 |
| Internet-supported versus face-to-face cognitive behavior therapy for depression | Not a systematic review |
| Internet-delivered psychological treatments for mood and anxiety disorders: a systematic review of their efficacy, safety, and cost-effectiveness | Publication year before 2015 |
| Fundamentals for future mobile-health (mHealth):A systematic review of mobile phone and web-based text messaging in mental health | Interventions that lack a synchronic component |
| The network approach to posttraumatic stress disorder: a systematic review | Not on the subject of e-health |
| Methods in predictive techniques for mental health status on social media: a critical review | Not a systematic review |
| Implementation of e-mental health interventions for informal caregivers of adults with chronic diseases: a protocol for a mixed-methods systematic review with a qualitative comparative analysis | It's a protocol |
| IoT-Based Services and Applications for Mental Health in the Literature | Interventions that lack a synchronic component |
| Evidence on virtual reality-based therapies for psychiatric disorders: Meta-review of meta-analyses | Systematic review not composed of primary studies |
| Internet-based self-help interventions for depression in routine care | Not a systematic review |
| Internet- and Mobile-Based Psychological Interventions: Applications, Efficacy, and Potential for Improving Mental Health: A Report of the EFPA E-Health Taskforce | Not a systematic review |
| EPA guidance on eMental health interventions in the treatment of posttraumatic stress disorder (PTSD) | Not a systematic review |
| Facebook-based social support and health: A systematic review | Other target population |
| Mobile Apps for the Management of Comorbid Overweight/Obesity and Depression/Anxiety: A Systematic Review | Other target population |
| Internet-Delivered Cognitive Behavioural Therapy for Major Depression and Anxiety Disorders: A Health Technology Assessment | Duplicate |
| Evidence of benefit from telemental health applications: a systematic review (Structured abstract) | Publication year before 2015 |
| Mobile Apps for Mental Health Issues: Meta-Review of Meta-Analyses | Systematic review not composed of primary studies |
| Is there a role for a web-based mental health and wellbeing self-management resource for use during pregnancy? A systematic review | Not a systematic review |
| Game-based digital interventions for depression therapy: A sytematic review and meta-analysis | Publication year before 2015 |
| A meta-analysis on the relationship between self-reported presence and anxiety in virtual reality exposure therapy for anxiety disorders | Publication year before 2015 |
| Mobile App Tools for Identifying and Managing Mental Health Disorders in Primary Care | Not a systematic review |
| Internet-based interventions for the prevention and treatment of depression in people living in developing countries: A systematic review | Other target population |
| Internet-Based Psychotherapy for Adult Depression: What About the Mechanisms of Change? | Interventions that lack a synchronic component |
| Online Intervention to Modify Interpretation Biases in Depression | It's a protocol |
| Implementations of Virtual Reality for Anxiety-Related Disorders: Systematic Review | Interventions that lack a synchronic component |
| Online and social networking interventions for the treatment of depression in young people: a systematic review | Publication year before 2015 |
| Online interventions for depression and anxiety - a systematic review | Publication year before 2015 |
| Mobile Mental Health: A Review of Applications for Depression Assistance | Systematic review not composed of primary studies |
| Help from the App Store?": A systematic review and evaluation of apps for use in depression | Duplicate |
| Help from the App Store?': A Systematic Review of Depression Apps in German App Stores | Systematic review not composed of primary studies |
| Mobile phone use and mental health-a review | Not a systematic review |
| Internet-Based Acceptance and Commitment Therapy: A Transdiagnostic Systematic Review and Meta-Analysis for Mental Health Outcomes | Other target population |
| Methodological challenges in randomized controlled trials on smartphone-based treatment in psychiatry: Systematic review | Other target population |
| Online psychological interventions to reduce symptoms of depression, anxiety, and general distress in those with chronic health conditions: A systematic review and meta-analysis of randomized controlled trials | Interventions that lack a synchronic component |
| A meta-analysis: Internet mindfulness-based interventions for stress management in the general population | Interventions that lack a synchronic component |
| Enlight: A Comprehensive Quality and Therapeutic Potential Evaluation Tool for Mobile and Web-Based eHealth Interventions. | Other target population |
| Internet-Based Cognitive Behavioral Therapy to Reduce Suicidal Ideation: A Systematic Review and Meta-analysis. | Interventions that lack a synchronic component |
| Telemental Health in Low- and Middle-Income Countries: A Systematic Review | Interventions that lack a synchronic component |
| TeleMental Health: Standards, Reimbursement, and Interstate Practice | Not a systematic review |
| The state of mental digi-therapeutics: A systematic assessment of depression and anxiety apps available for Arabic speakers | Not a systematic review |
| The use of technology in the clinical care of depression: An evidence map | Not a systematic review |
| A systematic review of online interventions for mental health in low and middle income countries: a neglected field | Interventions that lack a synchronic component |
| Telehealth Interventions Delivering Home-based Support Group Videoconferencing: Systematic Review | Other target population |
| PNS45 DETERMINANTS OF THE COST-EFFECTIVENESS OF TELEMEDICINE: RESULTS FROM A SYSTEMATIC REVIEW AND MULTIVARIABLE ANALYSIS | Not a systematic review |
| A systematic review of Internet-based supportive interventions for caregivers of patients with dementia | Publication year before 2015 |
| Virtual reality and its applications in mental disorders: A review | Systematic review not composed of primary studies |
| Virtual reality in psychiatric disorders: A systematic review of reviews | Systematic review not composed of primary studies |
| Telehealth and indigenous populations around the world: a systematic review on current modalities for physical and mental health | Other target population |
| Telemedicine: A systematic review of economic evaluations | Other target population |
| Virtual reality exposure in anxiety disorders: impact on psychophysiological reactivity | Publication year before 2015 |
| Psychological or educational eHealth interventions on depression, anxiety or stress following preterm birth: a systematic review | Other target population |
| Serious games for the treatment or prevention of depression: A systematic review | Publication year before 2015 |
| Therapist-supported online cognitive behavior therapy for adult anxiety | Not a systematic review |
| Technology-based cognitive training and rehabilitation interventions for individuals with mild cognitive impairment: A systematic review | Interventions that lack a synchronic component |
| Systematic review of research investigating psychotherapy and information and communication technologies | Other target population |
| The use of technology for mental healthcare delivery among older adults with depressive symptoms: A systematic literature review | Interventions that lack a synchronic component |
| Promises and risks of web-based interventions in the treatment of depression | Not a systematic review |
| A systematic review of technology-assisted interventions for co-morbid depression and substance use | Other target population |
| The Utilization of Technological Innovations to Support College Student Mental Health: Mobile Health Communication | Other target population |
| Using web-based interventions to support caregivers of patients with cancer: A systematic review | Systematic review not composed of primary studies |
| A systematic review and economic evaluation of computerised cognitive behaviour therapy for depression and anxiety (Structured abstract) | Publication year before 2015 |
| Smartphone applications for depression: a systematic literature review and a survey of health care professionals’ attitudes towards their use in clinical practice | Interventions that lack a synchronic component |
| A systematic review and meta-analysis of the efficacy of internet-delivered behavioural activation | Duplicate |
| Rationale and design of a systematic review: Effectiveness and acceptance of technology-based psychological interventions in different clinical phases of depression management | It's a protocol |
| Telemental health care, an effective alternative to conventional mental care: A systematic review | Other target population |
| Use of Virtual Reality for the Management of Anxiety and Pain in Dental Treatments: Systematic Review and Meta-Analysis | Retracted |
| Tailored Web-based interventions for pain: Systematic review and meta-analysis | Interventions that lack a synchronic component |
| The Use of Culturally-Tailored Telehealth Interventions in Managing Anxiety and Depression in African American Adults: A Systematic Review | Interventions that lack a synchronic component |
| Psychotherapeutic applications of mobile phone-based technologies: A systematic review of current research and trends | Interventions that lack a synchronic component |
| Unlocking smartphone potential in health care by providing smartphones to patients: A systematic review | Not a systematic review |
| Transdiagnostic computerised cognitive behavioural therapy for depression and anxiety: A systematic review and meta-analysis | Interventions that lack a synchronic component |
| Working alliance and outcome effectiveness in videoconferencing psychotherapy: A systematic review and noninferiority meta-analysis | Other target population |
| Psychosocial telephone interventions for patients with cancer and survivors: A systematic review | Publication year before 2015 |
| Smartphone applications for the treatment of depressive symptoms: A meta-analysis and qualitative review | Unable to retrieve |
| Web-based interventions for youth internalizing problems: a systematic review | Publication year before 2015 |
| User Acceptance of Computerized Cognitive Behavioral Therapy for Depression: Systematic Review | Interventions that lack a synchronic component |
| A systematic review of gamification in e-Health | Other target population |
| Technology-based interventions for mental health support after stroke: A systematic review of their acceptability and feasibility | Interventions that lack a synchronic component |
| A systematic review and meta-analysis of third-wave online interventions for depression | Interventions that lack a synchronic component |
| A Review Article on Internet-based Psychological Interventions in Primary Care. What is the Global Experience? How Reliable are Results from RCTs? Lessons Learned from the European, US and Australian Case Studies | Interventions that lack a synchronic component |
| What are the facilitators and barriers for the uptake and adherence to digital therapies for depression in adults? a systematic review of qualitative literature | Unable to retrieve |
| Telephone-administered psychological interventions for depression: A systematic review and meta-analysis | Systematic review not composed of primary studies |
| Toward the Design of Evidence-Based Mental Health Information Systems for People With Depression: A Systematic Literature Review and Meta-Analysis | Other target population |
| Systematic review of patients' participation in and experiences of technology-based monitoring of mental health symptoms in the community | Systematic review not composed of primary studies |
| Standalone smartphone apps for mental health—a systematic review and meta-analysis | Interventions that lack a synchronic component |
| The relationship between persuasive technology principles, adherence and effect of web-Based interventions for mental health: A meta-analysis | Not a systematic review |
| A Systematic Review and Meta-Analysis of Applicability of Web-Based Interventions for Individuals with Depression and Quality of Life Impairment | Interventions that lack a synchronic component |
| Web-based interventions to improve mental health in home caregivers of people with dementia: Meta-analysis | Interventions that lack a synchronic component |
| Technology mediated therapies and benefits: The influence of cybersickness and presence on the effectiveness of virtual reality exposure therapy to treat anxiety disorders: A meta-analysis | Unable to retrieve |
| Internet-delivered psychological interventions for clinical anxiety and depression in perinatal women: a systematic review and meta-analysis | Other target population |
