# Supplementary material for: Development of a Framework for the Implementation of Synchronous Digital Mental Health: Realist Synthesis of Systematic Reviews
Source: JMIR Ment Health. 2022 Mar 29;9(3):e34760. doi: 10.2196/34760 (PMC9006141; doi:10.2196/34760)
Supplement: Multimedia Appendix 4 [file mental_v9i3e34760_app4.docx]

**Supplementary Material 4. Characteristics of the included studies.**

| First author and year | Total or subgroup | Main characteristics of participants included in studies | Study design of primary studies | Number of studies included | Main objective | Quality assessment (Tool) | Electronic health interventions | Main findings |
| --- | --- | --- | --- | --- | --- | --- | --- | --- |
| Ahern, E. et al. (2018) [18] | Subgroup | The oCBT group (intervention) have females (54.92%) and males (22.31%), and mean age was 42.73 years. | RCTs | 30 | To assess the clinical and economic evidence for the use of oCBT for major depressive disorder. | Checklist items from Drummond and Jefferson Quality Assessment Scale | oCBT | Supported (guided) oCBT shows much promise as a cost-effective treatment in an adult population with major depressive disorder, at least in the short-term. oCBT can promote the accessibility of effective and affordable treatment, and therefore shows much promise to alleviate the psychological and economic costs of depression. Although oCBT was often shown to be more costly, the QALY improvements following oCBT tended to be greater, making it the favorable treatment at a Willingness to Pay threshold of £20,000/ QALY in a UK setting (56% likelihood of cost-effectiveness vs. treatment as usual), €30,000/ QALY in a Dutch setting (52% likelihood of cost-effectiveness vs. waiting list control), and at a Willingness to Pay threshold of $95,000/ QALY in an Australian setting (>95% likelihood vs. WLC). |
| Carlbring, P. et al. (2018) [33] | Subgroup | Adults | RCTs | 20 | To reinvestigate the efficacy of ICBT compared to face-to-face CBT for psychiatric and somatic disorders, considering studies published in the past four years. | Cochrane risk-of-bias tool | ICBT | The ICBT and face-to-face CBT are equally effective in treating social anxiety disorder, panic disorder, depressive symptoms, body dissatisfaction, insomnia, tinnitus, male sexual dysfunction, spider phobia, snake phobia, and fibromyalgia. |
| Castro, A. et al. (2020) [34] | Total | Adult participants (aged 18 and over) with major depression diagnosed using a structured clinical interview conducted according to internationally recognized standards (e.g., ICD-10, DSM-V) or significant (moderate to severe) depressive symptoms established using a validated screening measure (e.g., Patient Health Questionnaire -9), Beck Depression Inventory) | RCTs | 10 | To evaluate the effectiveness of telephone-administered psychotherapy for depression in adults when compared to control conditions or other active treatments and to determine adherence to telephone-administered psychotherapy. | Cochrane risk-of-bias tool | Telephone based CBT | Available evidence suggests that telephone-delivered psychotherapy may be an effective strategy to reduce depression symptoms when compared to control conditions and shows an adequate treatment adherence. |
| Coughtrey, A. et al. (2016) [51] | Subgroup | Adults (18 years and older) who had received an intervention to reduce symptoms of depression and/or anxiety. | RCTs, non-RCTs, and uncontrolled studies (cohort studies, open trials, or one-group pretest-posttest designs) if they obtained quantitative outcome data at a minimum of two time points (pre- and post-intervention) and the outcome data were statistically analyzed | 14 | To find the effectiveness evidence-based psychological therapies for adults with depression and/or anxiety in reducing psychological symptoms when delivered over the telephone. | Effective Public Health Practice Project Quality Assessment Tool | Therapies delivered by telephone | Of the 14 studies included for review, 13 reported statistically significant reductions in symptoms of depression and anxiety following an evidence-based treatment delivered by telephone. The findings of this review suggest that evidence-based interventions delivered by telephone show promise in reducing symptoms of depression and anxiety. |
| Cuijpers, P. et al. (2019) [35] | Subgroup | No details | RCTs | 155 | To examine the most effective delivery format for CBT via a network meta-analysis. | Using 4 criteria of the Cochrane risk-of-bias tool: adequate generation of allocation sequence; concealment of allocation to conditions; prevention of knowledge of the allocated intervention; and dealing with incomplete outcome data. | Telephone-based CBT | The individual, group, telephone-administered, and guided self-help treatment formats have comparable effectiveness in the treatment of depression, and that telephone-administered effectiveness does not differ statistically significantly across formats. The effect sizes of these treatment formats compared with the care as usual control condition were moderate or large when compared with the waiting list control condition. Although guided self-help CBT was as effective as an individual, group, and telephone CBT, it was less acceptable than the other formats. The unguided self-help was statistically significantly less effective than that of individual, group, telephone, and guided self-help CBT. |
| Domhardt, M. et al (2018) [43] | Subgroup | Adults aged 18 years or above, who meet diagnostic criteria according to a relevant classification system (e.g. DSM-IV, DSM-5 or ICD-10) at least for one of the following anxiety disorders diagnosed at baseline: specific phobia, social anxiety disorder, panic disorder, agoraphobia, or generalized anxiety disorder | RCTs | 34 | Summarize, evaluate, and meta-analyze integrating research on intervention components of Internet- and mobile-based interventions for adult anxiety disorders. | Cochrane risk-of-bias tool | Therapy by e-mail, videoconference, text messages, via telephone | Guided interventions were significantly more efficacious and improved adherence when compared to completely unguided interventions. |
| Drago, A. et al. (2016) [50] | Subgroup | No details | RCTs | 14 | Non-inferiority meta-analysis of distance psychiatric counseling compared to the face-to-face setting. | Downs and Black check-list | Videoconferencing | The non-inferiority of remote psychiatric counseling was reported both for assessment and treatment. |
| Finley, B. et al. (2020) [52] | Total | No details | case studies retrospective quasi experimental match-control study | 9 | Do a systematic exploration of Telepsychiatry use among (psychiatric mental health advanced practice registered nurses) psychiatric mental health advanced practice nurse practitioners. | The Grading of Recommendations Assessment, Development and Evaluation | Telepsychiatry, defined as delivering mental health care at a distance using electronic audio and visual teleconferencing technology | The limited existent literature demonstrates that psychiatric mental health advanced practice nurse practitioners can feasibly and successfully practice telepsychiatry. Most published of telepsychiatry literature predominantly focuses on clinical services across pediatric populations and the rural and urban underserved. Academic partner ships with primary care, hospitals, shelters, and federal clinics implemented and disseminated successful telepsychiatry consultations and scheduled services, but it is unclear if these programs were sustainable long-term despite short-term feasibility. There were no randomized experimental trials yielded from this search, demonstrating a lack of original and high-quality research including psychiatric mental health advanced practice nurse practitioners. |
| Irvine, A. et al. (2020) [36] | Total | Outpatients with insurance coverage mostly students of workers, one study of patients with mild TBI and one of HIV-living patients | Experimental studies and observational studies | 15 | To establish what research evidence exists to support such claims about the interactional differences between telephone and face-to-face therapy. | Ad-hoc designed bias evaluation design based on: (1) Bias in the comparison of face-to-face and telephone therapy. (2) Outcome measurement. (3) Sample representativeness. | Counselling, CBT, brief CBT, Recovery-focused CBT, and trained in Solution Focused Therapy | The telephone is a convenient, reliable and virtually universal communication channel. Yet despite evidence of comparable clinical outcomes, adoption amongst services is challenged by practitioner ambivalence, embedded views and systems that favors face-to-face. The available evidence does suggest a lack of support for arguments that the telephone has a detrimental effect on interactional aspects of psychological therapy. |
| Josephine, K. et al (2017) [44] | Total | Adult samples with a reliably diagnosed depressive disorder (i.e. major depression and/or persistent depressive disorder/dysthymia) | RCTs | 19 | To summarize and critically evaluate the effectiveness of internet- and mobile-based interventions for depression in adults with a diagnosed depression. | Cochrane risk-of-bias tool | No details | All Internet- and mobile-based depression interventions investigated in this review are effective in reducing depression symptoms in patients with a depressive disorder diagnosis. Internet- and mobile-based depression interventions showed a large beneficial effect on depression severity at the end of treatment compared to a waitlist control. |
| Kampmann, I. et al (2016) [37] | Subgroup | Adults with minimum age of 18 years who met the criteria for diagnosis of social anxiety disorder. | RCTs | 37 | to evaluate the efficacy of technology assisted interventions for individuals with a diagnosis of social anxiety disorder | Cochrane risk-of-bias tool | Guided internet delivered CBT | Guided ICBT was effective in reducing social anxiety disorder complaints compared to passive control conditions at post assessment. This effect did not sustain 5 months after the study; however, implications of this finding are limited by the fact that only two studies were included. The medium effect of guided ICBT relative to active control conditions indicated that guided ICBT might have an advantage over the active control. |
| Lewis, C. et al (2017) [38] | Total | Adults aged 16 years or older required to meet full diagnostic criteria for PTSD according to DSM or ICD criteria, assessed by clinical interview or a validated questionnaire. | RCTs | 10 | To determine whether ICBT is an effective treatment for those who meet diagnostic criteria for PTSD. | Cochrane risk-of-bias tool | ICBT | ICBT was more effective in the reduction of PTSD than no intervention or treatment as usual post-treatment. However, only three studies reported follow-up data and there was no evidence that treatment gains had been maintained at follow-up of less than six months. There was also evidence for greater effect in a sub-group analysis of only therapist guided ICBT. There was evidence of a significant difference in dropout rates from the ICBT group compared with the wait list/usual care group. There was evidence that ICBT was more effective than waitlist/ treatment as usual/ minimal attention in the reduction of symptoms of depression and anxiety post-treatment and at follow-up of less than six months. There was also evidence that ICBT was more effective than waitlist/ treatment as usual/ minimal attention post-treatment in terms of improvement in quality of life. |
| Linde, K. et al (2015) [45] | Subgroup | Adults (18 years or older). Patients must have been recruited from a primary care setting (primary care clinics, private practices of general practitioners, internists or other non-psychiatrists providing primary care in the respective country). Included patients had to suffer from prevalent or incident unipolar depressive disorder. | RCTs | 30 | To systematically review and compare the available evidence for the effectiveness of pharmacological, psychological, and combined treatments for patients with depressive disorders in primary care | Cochrane risk-of-bias tool | Remote therapy provided online, by telephone | The differences between different types of psychological treatments are minor, and remote therapist-led, guided self-help, and minimal-contact approaches can yield effects similar to personalized face-to-face therapies. There are also hints that psychological therapies might be less effective for patients having minor depression and dysthymia than for patients with major depression. |
| Moulton-Perkins, A. et al (2020) [39] | Total | Adults (>=18 years) | RCTs, non-controlled interventions, qualitative | 12 | To describe current evidence about the feasibility, acceptability, safety, and efficacy of delivering Mindfulness Based Cognitive therapy /Mindfulness-Based Stress Reduction for group videoconferencing | The Effective Public Health Practice Project Quality Assessment Tool for Quantitative studies | Mindfulness Based CBT /Mindfulness-Based Stress Reduction for group videoconferencing | Mindfulness-Based CBT and Mindfulness-Based Stress Reduction via Group Videoconferencing appears effective for reducing psychological distress compared with inactive controls, with medium effect sizes evidenced. Effectiveness compared with active controls was less clear however, as were any effects on potential mechanisms of action. No studies were sufficiently powered to conduct non-inferiority analyses comparing intervention to in-person treatment. |
| Olthuis, J. et al (2015) [46] | Subgroup | Adults of over 18 years of age; no upper limit with a primary diagnosis of an anxiety disorders. | RCTs | 30 | To assess the effects of therapist-supported Internet cognitive-behavioral therapy on remission of anxiety disorder diagnosis and reduction of anxiety symptoms in adults as compared to waiting list control, unguided CBT, or face-to-face CBT. | Cochrane risk-of-bias tool | Online treatment modules with email support from therapists, therapist support by telephone, online discussion forum | The internet based cognitive-behavioral therapy is more efficacious than a waiting list, attention, information, or online discussion group only control in leading to clinically important improvement in anxiety, reducing anxiety symptoms (both disorder-specific and general), and improving quality of life. Results also generally showed no difference in outcomes following therapist-supported ICBT versus unguided CBT at post-treatment, though results are limited by low quality evidence due to a limited number of studies (that is, imprecision). Moreover, results suggest that therapist-supported ICBT may not be significantly different from face-to-face group and individual CBT in treating anxiety disorders. Meta-analyses revealed no significant differences in clinically important improvement in anxiety or reduction in anxiety symptoms (both disorder-specific and general) at posttreatment or follow-up for these two interventions. |
| Olthuis, J. V. et al (2016) [47] | Subgroup | Military samples general population or clinical samples, including parents of children on cancer treatment, stem cell transplant patients, and women following childbirth. Thirteen of the included studies required participants meet PTSD criteria. The remaining six studies included some or all participants who met criteria for subclinical PTSD. | RCTs | 19 | To comprehensively review the outcomes from therapist-guided, distance-delivered interventions for PTSD | Cochrane risk-of-bias tool | Intervention delivered via videoconferencing, Internet-delivered interventions with telephone or email support, intervention supplemented with telephone support. | The videoconferencing seems to be comparable to face-to-face treatment delivery, at least for PTSD outcomes, further research into the comparability of these two interventions for depression outcomes, and for long-term follow-up is needed as the current results suggest an inferiority of videoconferencing in these areas. |
| Pasarelu, CR. et al (2016) [40] | Total | Adult participants (aged 18 years or older) that had either symptoms of anxiety and/or unipolar depression or a primary diagnosis of anxiety and/or unipolar depression with comorbid anxiety and/or unipolar depression. | RCTs | 19 | To provide a meta-analysis of the published studies on transdiagnostic and tailored ICBT for adult patients with symptoms of anxiety and/or depression or with a primary diagnosis of anxiety and/or unipolar depression with comorbid anxiety and/ or unipolar depression. | Cochrane risk-of-bias tool | Internet-delivered transdiagnostic and tailored CBT | Internet-delivered transdiagnostic and tailored CBT are promising interventions that have a moderate-to-large effect size on symptoms of anxiety and depression. Also, the effect of such interventions on quality-of-life assessments is moderate. The adherence (time completed the entire problem) varied from 32 to 89%. Treatment length did not moderate anxiety and quality of life outcomes. However, in the case of depression outcomes, longer treatments (more than six modules) were associated with larger effect size. The amount of time a therapist spent with each patient was not associated with better outcomes, even though this index varied from 19 min or 150 min. |
| Proctor, B. J. et al (2018) [48] | Subgroup | People with Multiple Sclerosis in which a telephone-delivered psychological therapy was compared to a control. | RCTs | 11 | To review the evidence for the effectiveness of telephone-psychotherapy on psychological outcomes in people with Multiple Sclerosis compared to those receiving no treatment, standard care or other control, on psychological and physical outcomes. | Cochrane risk-of-bias tool | Telephone-psychotherapy | A small non-significant treatment effect was found on depression favoring the control group. There is some mixed and poor-quality evidence that suggest that providing telephone-psychotherapy to people with Multiple Sclerosis has small-to-moderate benefits on depression in the short-term. Meta-analysis found a moderate effect of the intervention on depression. The designs of the RCTs were heterogeneous. All studies had at least a high or unclear risk of bias. |
| Rees, C. S. et al (2015) [41] | Subgroup | No details | Controlled, Case study, Uncontrolled case series, Uncontrolled | 20 | To synthesize the current literature on the effectiveness of videoconference delivered therapy for anxiety disorders. | No details | Exposure therapy for PTSD, cognitive processing therapy for PTSD, prolonged exposure for PTSD, group cognitive processing therapy–cognitive, group CBT, CBT for PTSD, prolonged exposure, behavioral activation and therapeutic exposure, cognitive processing therapy–cognitive, CBT. | Not reported |
| Sunjaya, A. et al (2020) [42] | Total | No details | RCTs, case-control and cohort study design | 15 | To evaluate the potential of telemedicine as an alternative solution to bridge the barriers towards better PTSD care in Indonesia and other countries. | No details | Teleconference, online CBT | Various studies have shown telepsychiatry as an effective and efficient way to manage PTSD. The quality of care given by telepsychiatry both through video conferencing as well as web and application based is comparable to that of face-to-face therapy, although most studies were from developed nations. Patient satisfaction, quality of doctor patient relationship also remains high with therapeutic time comparable to that of face-to-face therapy. Countries especially with low patient and mental health professional ratio should therefore be encouraged to develop telepsychiatry systems to manage PTSD. |
| Turgoose, D. et al (2017) [49] | Total | Veterans with PTSD |  | 41 | To systematically review findings from studies using tele-therapy interventions to treat PTSD in military veterans, in order to provide a more robust overview of lessons learned so far from using tele-therapy in this population, and to inform the use of such interventions in the future. | Quality Assessment Tool for Quantitative Studies | Videoconferencing, telephone-based counselling | Eighteen studies looked at the clinical effectiveness of teletherapy interventions. Of these, 15 used a control, typically the same form of intervention but in person. All of these studies reported that tele-therapy was associated with significant reductions in PTSD symptoms. Of these 18 studies, 12 reported comparisons between tele-therapy and in-person interventions using non-inferiority analyses, with nine concluding that tele-therapy was as effective as in person therapy. |

Note: CBT: Cognitive behavioral therapy; DSM: Diagnostic and Statistical Manual of Mental Disorders; ICBT: Internet-based cognitive behavioral therapy; ICD-10: International Statistical Classification of Diseases and Related Health Problems; oCBT: Online cognitive behavioral therapy; RCTs: Randomized-controlled trials; PTSD: Post-traumatic stress disorders. QALY: Quality adjusted life years
