# Supplementary material for: Development of a Framework for the Implementation of Synchronous Digital Mental Health: Realist Synthesis of Systematic Reviews
Source: JMIR Ment Health. 2022 Mar 29;9(3):e34760. doi: 10.2196/34760 (PMC9006141; doi:10.2196/34760)
Supplement: Multimedia Appendix 5 [file mental_v9i3e34760_app5.docx]

Confidence in cumulative evidence assessed with the Confidence in Evidence from Reviews of Qualitative Research (CERQual) approach.

| Summary of review finding | References | Methodological limitations | Coherence | Data adequacy | Relevance | CERQual assessment of confidence in the evidence |
| --- | --- | --- | --- | --- | --- | --- |
| Diverse synchronous digital interventions (SDIs) in mental health effectively reduce symptoms and readmissions, similar to in-person interventions. However, these SDIs differ and there is not a clear type of SDI that is better than others. | 19 studies: [31-39,41-50] | Serious concerns regarding methodological limitations: one study with medium confidence, one study with low confidence, and 17 studies with very low confidence due to lack of exhaustive bibliographic search, absence of review protocol, and no justification for meta-analysis methods. | No or very minor concerns. All reviews found positive or similar results for diverse SDIs compared to usual-care interventions or waiting list. | No or very minor concerns. These reviews included more than 100 individual studies. Most of these studies assessed the effects of these SDIs in mental health. | Minor concerns since the review finding is based on systematic reviews that included all recently published data. However, there is still lack of representation of low- and middle-income countries | Low confidence |
| SDIs in mental health reach underserved populations, since they do not require the *physical presence* of a therapist nor the patient, thereby tackling geographic barriers posed by in-person therapy. However, these interventions could create new challenges, including access and literacy of technology and patient security *(Hypothesis 1).* | 13 studies: [31,32,36,38,40-42,45-49,51] | Serious concerns regarding methodological limitations: one study with medium confidence and 12 studies with very low confidence due to lack of exhaustive bibliographic search, absence of review protocol, and incorrect methods for selection and data extraction processes. | Minor concerns since these reviews agreed about the benefits of SDIs in mental health, but not all of them assessed the possible new barriers for the underserved population. | Minor concerns since most of these reviews mentioned the tackling of geographic barriers. However, they offered superficial information about this phenomenon, according to few primary studies. | Minor concerns since the review finding is based on systematic reviews that included all recently published data. However, there is still lack of representation of low- and middle-income countries | Very low confidence |
| SDIs in mental health can be successfully *delivered by nonspecialists, and therefore they are more cost-effective* to implement in health services. However, there is a need for training and supervision for human resources, and this can create distrust in some health personnel (*Hypothesis 2*) | 12 studies: [31,33,35,37-42,48,49,51] | Serious concerns regarding methodological limitations: one study with low confidence and 11 studies with very low confidence due to lack of exhaustive bibliographic search, absence of review protocol, and incorrect interpretation of risk of bias of individual studies. | Moderate concerns since some reviews acknowledged the higher cost of SDIs, but not all of them agreed about the cost-effectiveness of these interventions. | Minor concerns since most of these reviews mentioned the tackling of geographic barriers. However, they offered superficial information about this phenomenon, according to few primary studies. | Minor concerns since the review finding is based on systematic reviews that included all recently published data. However, there is still lack of representation of low- and middle-income countries | Very low confidence |
| SDIs in mental health *are acceptable by patients and show good results in satisfaction,* because they require less need of disclosure and provide more privacy, comfortability, and participation, enabling the establishment of rapport with the therapist (*Hypothesis 3).* | 10 studies: [31,33,35,38,40-42,46,48,51] | Serious concerns regarding methodological limitations: all 10 studies with very low confidence due to lack of exhaustive bibliographic search, absence of review protocol, and incorrect assessment and interpretation of heterogeneity and risk of bias of individual studies. | Moderate concerns since some reviews also highlighted some barriers for these SDIs, which could influence the patients’ perceptions about the interventions | Minor concerns since most of these reviews mentioned the tackling of geographic barriers. However, they offered superficial information about this phenomenon, according to few primary studies. | Minor concerns since the review finding is based on systematic reviews that included all recently published data. However, there is still lack of representation of low- and middle-income countries | Very low confidence |
